# GPR137-RAB8A activation promotes ovarian cancer development via the Hedgehog pathway

**DOI:** 10.1186/s13046-025-03275-0

**Published:** 2025-01-24

**Authors:** Chao Tang, Lin Li, Chongying Zhu, Qiang Xu, Zihao An, Shouying Xu, Chao Lin

**Affiliations:** 1 National Clinical Research Center for Child Health of Children’s Hospital, Zhejiang University School of Medicine, No. 3333, Binsheng Rd, Hangzhou, 310052 People’s Republic of China; 2 Department of Urology, Third Affiliated Hospital, Naval Medical University, Shanghai, 201805 China; 3 The Department of Obstetrics and Gynecology, Ruijin Hospital, Shanghai Jiaotong University School of Medicine, 197 Ruijin 2 Road, Shanghai, 200025 China; 4 Department of Neurosurgery, Children’s Hospital, Zhejiang University School of Medicine, Hangzhou, China

**Keywords:** GPR137, RAB8A, Hedgehog, Ovarian cancer

## Abstract

**Background:**

Ovarian cancer (OC) progression is one of the commonest cause of female cancer death. While treatments in clinic includes primary surgery and targeted chemotherapy, curative and survival trends in OC have not significantly improved. Thus, further investigation of the mechanisms regarding OC carcinogenesis and discovery of novel targets is of great importance.

**Methods:**

Human ovarian tissue specimens, RNA sequencing, GEPIA database and bioinformatics analyses were used to analyze the gene correlation, and to identify and validate potential downstream candidates. The biological effects of GPR137-RAB8A-Hedgehog(HH) were investigated using in vitro and in vivo models and methods including qRT-PCR, RNA stability assay, RNA immunoprecipitation assay, GLI-luciferase reporter assay, nucleo-cytoplasmic separation assay, membrane-cytoplasmic separation assay, western blot, co-immunoprecipitation, immunofluorescence staining, cell counting kit-8 assay, wound healing assay, matrigel invasion assay, colony formation assay, xenografts assay, in situ transplantation tumor model of ovarian cancer in nude mice, and immunohistochemistry staining.

**Results:**

GPR137 expression was significantly higher in collected clinical OC tissues, compared with the adjacent normal tissues. Consistently, suppression of GPR137 inhibited human SK-OV-3 and A2780 OC cell proliferation, migration, invasion, and colony formation, whereas overexpression of GPR137 in human OC HO8910 cell exerted the opposite effects on cell biological behaviors. Mechanistically, *RAB8A* was identified as a downstream target of *GPR137*, and GPR137 promotes *RAB8A* expression by promoting *RAB8A* mRNA stability. By RNA-sequencing and experiments in vitro using multiple ovarian cancer cell models as well as in vivo using subcutaneous xenografts assay and in situ transplantation ovarian cancer model in nude mice, we further demonstrated that RAB8A positively mediated OC progression through activating HH signaling pathway by disassociating the protein–protein complex formation of GLI and SuFu (Suppressor of Fused), which reciprocally enhanced GPR137 activity, forming a regulation loop between HH signaling and GPR137.

**Conclusions:**

Collectively, this study depicts the role of GPR137-RAB8A-HH cascade in the development of OC, deepening our understanding of tumor biomechanics regarding OC progression and providing novel targets for OC therapy in future.

**Supplementary Information:**

The online version contains supplementary material available at 10.1186/s13046-025-03275-0.

## Introduction/background

Ovarian cancer (OC) is one of the leading cause of gynecological cancer-related mortality in women worldwide [1–3]. Despite the advances in treatments with primary surgery and targeted chemotherapy recently, the prognosis has only marginally improved because of the relapse and chemotherapy resistance in clinic [4, 5]. In addition, metastasis has emerged to be a major difficulty of OC therapy, which fails to provide satisfactory outcomes [6, 7]. Therefore, thorough understanding of the pathogenically molecular mechanisms regarding with OC occurrence and progression holds great significance to discover and develop new treatment strategies and ameliorate more effectually the overall prognosis of the patients with OC.

G protein-coupled receptor 137 (GPR137, also known as C11orf4, GPR137A or TM7SF1L1), is an orphan member in the family of cell surface mediators of signal transduction [8, 9]. Located in 11cen-q13.1, *GPR137* was initially discovered through custom searching the GenBank genomic databases based on known sequences coding G protein-coupled receptor (GPCR). The *GPR137* gene encodes a peptide containing four trans-membrane domains in the cytoplasm, with signal peptides at both the N-termini and the C-termini. Despite that GPR137 *transcription* was primarily discovered in the human hippocampus [10], GPR137 expression has been later revealed to be ubiquitously presented in other organs and tissues, including the hematological system and reproductive system [11–13], indicating the potential involvement of GPR137 in multiple physiological progressions. Recently, it has been reported that GPR137 also plays a crucial role in tumor progression and metastasis, including ovarian cancer (OC), and GPR137 is found to be highly expressed in clinical OC tissues [14], providing with possible prognostic value of GPR137 in OC. However, the role of GPR137 in the progression of OC remains incompletely understood.

Therefore, we herein aim to investigate whether and how GPR137 exerts influence on OC progression. We confirmed that GPR137 expression was significantly higher in collected clinical OC tissues, compared with the adjacent normal tissues. Consistently, suppression of GPR137 inhibited human OC cells (including SK-OV-3 and A2780) proliferation, migration, invasion, and colony formation, whereas overexpression of GPR137 in human OC HO8910 cell exerted the opposite effects on cell biological behaviors. Mechanistically, through multiple experimental analyses, *RAB8A* (Ras-related protein Rab-8A, a member of the Rabs family) [15, 16] was identified as a downstream gene of *GPR137* and GPR137 promotes RAB8A expression by promoting *RAB8A* mRNA stability. By RNA-sequencing, bioinformatics as well as multiple experiments, we further demonstrated that RAB8A positively mediated OC progression through activating Hedgehog (HH, an important morphogen in controlling embryonic development and carcinogenesis [17, 18]) signaling pathway by disassociating the protein–protein complex formation of GLI and SuFu (Suppressor of Fused, a critical repressor component in HH signaling [19]), which reciprocally enhanced GPR137 activity, forming a regulation loop between HH signaling and GPR137. Collectively, this study deepens our understanding of tumor biomechanics of OC progression and provides novel targets for OC therapy in future.

## Materials and methods

### Human ovarian tissue specimens

Totally 10 pairs of high-grade serous ovarian carcinoma tissues and adjacent non-tumor tissues were obtained from patients undergoing surgery at Ruijin Hospital, Shanghai Jiaotong University School of Medicine from 2022 to 2024. The fresh tissue samples were collected immediately after surgery and stored in preservation buffer at -80℃. Patients didn’t receive chemotherapy or radiotherapy before operation. Informed consent was obtained from each patient, and the use of clinical samples in this study was approved by the Ruijin Hospital, Shanghai Jiaotong University School of Medicine institutional review board.

### Cell lines and cell culture

Human ovarian cancer cell lines SK-OV-3 and A2780 cells, human immortalized ovarian surface epithelial cell line IOSE80, and human embryonic kidney 293 T (HEK293T) cell line were obtained from the American Type Culture Collection (ATCC, Manassas, USA). Human ovarian cancer cell line HO8010 was purchased from the Type Culture Collection Centre of Chinese Academic of Science (Shanghai, China). Cells were maintained in high glucose DMEM (Life Technologies, Inc., Grand Island, NY) supplemented with 10% (v/v) Fetal bovine serum (FBS, Life Technologies, Inc., Grand Island, NY) and 1% (v/v) penicillin–streptomycin solution (Beyotime Biotechnology, Shanghai, China) in a cell incubator (Thermo Scientific) at 37 °C with 5% CO_2_ as described previously [8, 20–22].

### Oligonucleotides, plasmids, viruses and infections

The primers for quantitative Real Time PCR (qRT-PCR) were as follows:

- human *GPR137*-F-5’-ACCTGGGGAACAAAGGCTAC-3’;
- human *GPR137*-R-5’-TAGGACCGAGAGGCAAAGAC-3’;
- human *RAB8A*-F-5’-CTACGACATCACCAACGAGAAG-3’;
- human *RAB8A*-R-5’-CATCACACTTGTTCCCGAGTAT-3’;
- human *GLI1*-F-5’-CCACGGGGAGCGGAAGGAG-3’;
- human *GLI1*-R-5’-ACTGGCATTGCTGAAGGCTTTACTG-3’;
- human *PTCH1*-F-5’-ACAAACTCCTGGTGCAAACC-3’;
- human *PTCH1*-R-5’-CTTTGTCGTGGACCCATTCT-3’;
- human *GAPDH*-F-5’-CCTCAACTACATGGTTTACATGTTCC-3’;
- human *GAPDH*-R-5’-GAAGATGGTGATGGGATTTCCATTG-3’.

The human GPR137 and RAB8A lentiviral expression vectors, pCDH-CMV-GPR137-EF1-Puro and pCDH-CMV-RAB8A-EF1-Puro, and GPR137-shRNA and RAB8A-shRNA-expressing lentiviral vectors, pLV3-U6-GPR137(human)-shRNA-Puro and pLV3-U6-RAB8A(human)-shRNA-Puro, were constructed by Mr. Qiang Xu, and the pCDH-CMV-MCS-EF1-Puro empty vector and the pLV3-U6-shRNA-Puro vector containing a scrambled shRNA sequence were used as a control, respectively. Lentiviruses were generated as described previously [23], and the lentiviruses-containing supernatants with the titers greater than 1 × 10^6^ cfu/ml was applied for infection of human SK-OV-3, A2780, or HO8910 cells in the presence of 8 μg/ml polybrene (Sigma, St. Louis, MO, USA).

### Antibodies and chemicals

GPR137 (bs-16270R, 1:1000), RAB8A (bs-6176R, 1:1000), GLI2 (bs-11564R, 1:1000), and PCNA (bs-2007R, 1:1000) antibodies were from Bioss (Beijing, China). Flag (M185-3, 1:10000) antibody was from MBL (Beijing, China), and antibodies for GLI1 (sc-20687, 1:1000), GLI3 (sc-74478, 1:1000), SuFu (sc-137014, 1:1000), IFT88 (sc-84318, 1:1000), KIF3A (sc-376680, 1:1000), MMP-2 (sc-13595, 1:1000), MMP-9 (sc-21733, 1:1000), GFP (sc-9996, 1:5000), EZRIN (sc-58758, 1:1000), GAPDH (sc-32233, 1:2000) and normal mouse IgG (sc-2025) were from Santa Cruz Biotechnology (Santa Cruz, CA, USA). PTCH1 (#2468, 1:1000) antibody, and normal rabbit IgG (#2729) were purchased from Cell Signaling Technology (Danvers, MA, USA). Ki67 antibody (ER1706-46, 1:200) was from Huabio (Hangzhou, China), and αTubulin antibody (AF5012, 1:1000) was from Beyotime Institute of Biotechnology (Shanghai, China). Alexa555-conjugated secondary antibody was from Life Technology. DAPI (2-(4-Amidinophenyl)-6-indolecarbamidine dihydrochloride, #C1002) was obtained from Beyotime Institute of Biotechnology (Shanghai, China). Recombinant human SHH protein (N-Shh, C24II, N-Terminus) was from R&D Systems (#1845-SH). Actinomycin D, SAG, GDC-0449, and GANT61 were purchased from Selleckchem (Shanghai, China).

### RNA isolation, reverse transcription and qRT-PCR

Total RNA was isolated from SK-OV-3, A2780, HO8910, and IOSE80 cells, or from fresh clinical tissues by using a TRIzol reagent (Takara Biotechnology Co., Ltd., Dalian, China) as per the manufacturer’s instructions. Briefly, a total of 5 μg RNA in a volume of 20 μl was reversely transcribed with a SuperScript III reagent (Life Technologies) and the oligo-(deoxythymidine) primer with incubation at 42 °C for 1 h. After cDNA synthesis, each reaction mixture was diluted with a volume of 80 μl Tris–EDTA buffer. qRT-PCR was subsequently conducted to examine the mRNA expression levels of human *GPR137*, *RAB8A*, *GLI1* and *PTCH1* using corresponding paired primers, respectively. The relative amounts of the target mRNA levels were normalized to the *GAPDH* levels, respectively, and the relative difference in mRNA levels was consequently calculated by 2^−∆∆Ct^ method as described previously [8]. Data were displayed as the relative expression of target genes in each group to the corresponding control group (vehicle, control, or scramble shRNA group) that was set as “1”.

### RNA stability assay

RNA stability assay was carried out as previously described [23]. Briefly, 5 × 10^5^ GPR137-shRNA-expressing or control scrambled sequence-expressing SK-OV-3 or A2780 cells were seeded in 3.5-cm cell culture plates and were treated with 10 μg/ml actinomycin D (AcD). Cells were subsequently harvested at time 0 h, 2 h, 4 h, and 8 h time points, and RNA was isolated and subjected to qRT-PCR as described above, with equal amount of RNA from the four time points provided to each RT reaction. In each group, RNA expression at different time points was normalized against that at the corresponding 0 h time point to calculate relative fold-enrichment.

### RNA immunoprecipitation (RIP) assay

RIP assay was conducted as described previously [23]. In brief, an actively growing SK-OV-3 cell monolayer was cross-linked with 1% formaldehyde, and then cross-linking was stopped by 0.25 M glycine. Cells were lysed in immunoprecipitation assay buffer RIPA containing 50 mM Tris (pH 7.5), 1% NP-40, 0.5% sodium deoxycholate, 0.05% SDS (sodium dodecyl sulfate), 1 mM EDTA, 150 mM NaCl, 40 units RNAsin, and protease inhibitors. Lysates were then sonicated, and insoluble material was removed by centrifugation and precleared with protein A Sepharose, followed by immunoprecipitation experiment with GPR137 antibody or corresponding IgG. The harvested immunoprecipitates were subsequently washed five times in high-stringency RIPA buffer containing 50 mM Tris (pH 7.5), 1% NP-40, 1% sodium deoxycholate, 0.1% SDS, 1 mM EDTA, 1 M NaCl, 1 M urea, 40 units RNAsin and protease inhibitors. Protein was then eluted with elution buffer containing 50 mM Tris (pH = 7), 5 mM EDTA, 10 mM DTT, 1% SDS, and cross-links were reversed at 70 °C for 45 min. RNA was extracted by TRIzol reagent (Takara Biotechnology Co., Ltd., Dalian, China) and treated with DNase I (DNA-free kit, Ambion), and bound *RAB8A* RNAs were consequently analyzed by qRT-PCR as described above.

### Transient transfection and GLI-luciferase (GLI-Luc) reporter assay

Transient transfections in cells were carried out by using Lipofectamine 2000 reagent (Invitrogen, Thermo Fisher Scientific) as per the manufacturer’s instructions. GLI-Luc reporter (containing 8 × GLI-binding sites) activities were determined by using the dual luciferase reporter assay (Promega) as per the manufacturer’s protocol as described previously [17]. A pRL/TK-luciferase reporter plasmid purchased from Promega was utilized as a second reporter for normalizing the results. The data were obtained by analyzing triplicated samples.

### Nucleo-cytoplasmic separation assay, membrane-cytoplasmic separation assay, western blot and co-immunoprecipitation (co-IP)

For nucleo-cytoplasmic separation assay, the nuclear and cytosolic fractions of HO8910 cells were prepared by using the nuclear and cytosol protein extraction kit from Beyotime Institute of Biotechnology (#P0027, Shanghai, China) according to the manufacturer's protocol as described previously [23]. For membrane-cytoplasmic separation assay, the membrane and cytosolic fractions of SK-OV-3 and A2780 cells were obtained by using the membrane and cytosol protein extraction kit from Beyotime Institute of Biotechnology (#P0033, Shanghai, China) according to the manufacturer's protocol as described previously [24]. Western blots were performed using standard protocols as described previously [9]. Briefly, total protein extracts were prepared, and protein concentrations were determined by using a standard Bradford assay. After that, a total of 50 μg protein was subjected to SDS-PAGE followed by a transfer onto PVDF (Polyvinylidene fluoride) membranes (Millipore, Bedford, MA). Membranes were incubated with target primary antibodies at 4 °C overnight followed by incubation in corresponding secondary antibodies (Beyotime Institute of Biotechnology) at room temperature for 2 h. The intensity of protein bands-derived signals was subsequently quantified using a NIH ImageJ software (ImageJ, http://rsb.info.nih.gov/ij/). Co-immunoprecipitation was conducted as described previously [25]. In brief, SK-OV-3, A2780 or HO8910 cells were harvested and lysed in IP lysis buffer (containing 100 mM NaCl, 50 mM Tris–HCl (pH 8.0), 5 mM EDTA, 1% Brij35, 2 mM Na3VO4, 10 mM NaF, 2 mM β-glycerophosphate and 2 mM PMSF), and were incubated with different antibodies or protein A/G plus agarose (sc-2003, Santa Cruz). The beads were next washed five times with the IP lysis buffer, and the immune complex was eluted with western blot sample buffer. Lysates and immunoprecipitates were subjected to western blot as described above.

### Immunofluorescence (IF) staining

IF staining was carried out using chamber slides (Nalge Nunc International, Naperville, IL) as described previously [23]. N-SHH-rp-treated SK-OV-3 cells were fixed in ice-cold methanol for 10 min and were permeabilized next with 0.1% TritonX-100 in PBS (PBST) for 10 min. After blocking with BSA solution, SK-OV-3 cell samples were incubated with a GPR137 primary antibody and subsequently with the fluorescent Alexa 555-conjugated secondary antibody. Nuclei were counterstained with DAPI at room temperature for 5 min. Sample slides were then analyzed by a laser scanning microscope (Zeiss, Germany).

### Cell proliferation by cell counting Kit-8 (CCK-8) assay

CCK-8 assay was conducted as per the manufacturer’s protocol (Yeasen, Shanghai, China) as described previously [8]. After treatment, SK-OV-3, A2780, or HO8910 cells were incubated with 10 μl CCK-8 reagent per well (96-well plate) for 2 h at 37 °C in the dark and the absorbance was subsequently measured at a wavelength of 450 nm using a micro-plate reader (Spark, Switzerland). Data were displayed as fold changes in each group relative to the corresponding control at 0 h.

### Wound healing assay

Wound healing assay was carried out as previously described [26]. In brief, SK-OV-3, A2780, or HO8910 cells (4 × 10^5^ cells) were seeded in six-well plate and were transfected with indicated overexpressing- and/or shRNA sequence-carrying plasmids. 24 h after transfection, cells were subjected to serum starvation for another 12 h. After rinsed with medium to remove unattached cells, the confluent layer of cells was scratched with a sterile 10 μl-tip to create an artificial wound. Cell migration to the wounded gap was subsequently monitored by microscopy after 48 h and the wound closure was analyzed using ImageJ software.

### Matrigel invasion assay

Invasive capacity of cells was measured using Matrigel (BD Biosciences, NJ)-coated Transwell inserts (6.5 μm, Costar, Cambridge) containing polycarbonate filters with 8 μm pores as detailed previously [8]. In brief, the mixture of Matrigel and medium with a volume ratio of 1:2 (totally 50 µl) was enclosed by each Transwell membrane. After transfected with indicated overexpressing- and/or shRNA sequence-carrying plasmids and cultured for 48 h, SK-OV-3, A2780, or HO8910 cells (2 × 10^5^) in a total of 200 µl volume of serum-free medium were seeded in the upper chamber, whereas 600 µl volume of medium with 10% FBS was added into the lower well. After 24 h-incubation, the non-invading cells that remained on the upper surface of the filter were removed slightly, and the cells that had passed through the filter and attached to the bottom of the membrane were then fixed in methanol at room temperature for 10 min and stained with 0.2% crystal violet for 5 min. Numbers of the invasive cells were counted randomly in six selected fields from triplicate chambers in each experiment. The migrated cells were counted under a light microscope, and data were displayed as fold changes in each group relative to the control.

### Colony formation assay

Colony formation assay was carried out as previously described [27, 28]. In brief, SK-OV-3, A2780, or HO8910 cells were infected with indicated lentiviruses that carrying overexpressing- and/or shRNA sequence. After 24 h, the cells were seeded in six-well plates at a density of 1000 cells/well in 2 ml culture medium per well. After two week-culture, colonies were fixed in methanol at room temperature for 10 min and then stained with 0.5% crystal violet in methanol or 0.25% Coomassie blue in methanol. Colonies were subsequently counted and photographed, and the colony numbers were consequently statistically analyzed.

### Xenografts assay

Female Balb/c-nu/nu mice (6-week-old) were rested for a week and were bred under pathogen-free conditions (temperature, 18–22 °C; humidity, 50–60%; 12/12-h light/dark cycle). Tumor-bearing mice model was established by subcutaneous inoculation with xenografts of human HO8910 cells (5 × 10^6^ cells in 100 µl volume of PBS per site) infected with control lentiviruses or RAB8A-overexpressing lentiviruses, or with human SK-OV-3 cells (5 × 10^6^ cells in 100 µl volume of PBS per site) infected with control lentiviruses carrying a scrambled sequence or lentiviruses carrying *GPR137*-shRNA sequence into the left armpit as described previously [20]. The body weights, and volumes (V) of the xenograft tumors were measured every 3 d in SK-OV-3 cell infected nude mice as follows: V = 0.5 × a × b^2^, where “a” indicates the long axis and “b” indicates the short axis. When the volume of one tumor reaches 800 mm^3^ at day 18 (18 d) after inoculation, all the mice were sacrificed, and the subcutaneous tumors were collected, weighed and used for further analysis. All animal care and handling procedures were approved by the Institutional Animal Care and Use Committee of Zhejiang University, and were performed according to the Guide for the Care and Use of Laboratory Animals (NIH Publication No. 85–23, revised 1996).

### In situtransplantation tumor model of ovarian cancer in nude mice

Female Balb/c-nu/nu mice (6–8 week-old, 18–20 g of body weight) were rested for a week and were bred under pathogen-free conditions (temperature, 18–22 °C; humidity, 50–60%; 12/12-h light/dark cycle). The experimental nude mice were fixed after anesthesia, the back skin was prepared and disinfected, a longitudinal incision of one cm was made from the center of the back to the right, and an incision of one cm was made on the upper edge of the iliac crest. A volume of 0.01 ml (3.0 × 10^6^ cells) of *RAB8A*-shRNA-expressing or control lentiviruses infected-A2780 cell suspension was injected into the deep part of the left ovary, compressed for one minute, and the injection hole was sealed with biological protein gel. The body weights of the mice were measured every 4 d, and when the volume of one tumor reaches 800 mm^3^ at day 28 (28 d) after inoculation, all the mice were sacrificed, and the ovarian tumors were collected, weighed and used for further analysis. All animal care and handling procedures were approved by the Institutional Animal Care and Use Committee of Zhejiang University.

### Immunohistochemistry (IHC) staining

IHC staining was conducted by using the Histostain-Plus Kit (Kangwei Reagents, Beijing, China) according to the manufacturer’s instructions as detailed previously [20]. In brief, paraffin-embedded xenografts tumor sections or human clinical OC sections of 4 μm-thickness were deparaffinized and rehydrated in xylene and a graded series of ethanol. After antigen retrieval in solution containing 10 mM sodium citrate and 10 mM citric acid, tissue sections were next incubated with 3% H_2_O_2_ in methanol to quench endogenous peroxidase followed by sequential incubation including with normal serum for 30 min at room temperature, with primary antibodies against GPR137, RAB8A, Ki67, or IgG at 4 °C overnight, and subsequently with HRP-labeled secondary antibody (Life Technologies) at room temperature for 30 min. The diaminobenzidine (DAB) solution was used for development of color, and the sections were counterstained with hematoxylin. The sections were consequently sealed with neutral resin and observed under the microscope. GPR137 expression and RAB8A expression was scored as previously described [9] based on the proportion of cells showing GPR137 or RAB8A IHC staining across three non-adjacent fields in each sample with the following criteria: 0 (no cell positive); 1 (< 50% cells weakly positive); 2 (< 50% cells intensely staining); 3 (≥ 50% cells weakly positive); 4 (≥ 50% cells intensely staining).

### RNA-Sequence (Seq) analysis

A minimum of 3 μg of total RNA from RAB8A-overexpressing HO8910 cells was oligo (dT) selected using the Dynabeads mRNA purification kit (Invitrogen). Then, the mRNA was isolated from total RNA and was next fragmented into short fragments with a fragmentation buffer (Ambion), and double-stranded cDNA was subsequently synthesized with these obtained short fragments as templates. Next, the generated cDNA was end-repaired, ligated to Illumina adapters, size selected on agarose gel (approximately 250 bp) and PCR amplified. The cDNA library was then sequenced on an Illumina HiSeq 2000 sequencing platform (Berry Genomics). The Stringtie software was applied for predicting the transcripts of all samples, and the RSEM software was used to call bowtie2 for comparison results. The number of reads aligned to each transcript for each sample was obtained, and the gene expression levels for each transcript were estimated as the number of reads per kilo-base of exon model per million mapped reads (RPKM) using only uniquely mapped reads in exonic regions. After a comprehensive analysis of gene expression levels, differential gene analysis between sample groups was performed. A gene is considered significantly differentially expressed if its expression differs between samples from the two groups, control group and RAB8A-overexpression group, with the log_2_(Fold Change) > 1 or < -1, and the *p* value < 0.05 as calculated by Cufflinks. Volcano plots and cluster heatmaps were drawn for visually displaying the differentially expressed genes between the two groups, followed by Gene Ontology (GO, http://www.geneontology.org/) enrichment analysis of GO terms with false discovery rate (FDR) ≤ 0.05 using hyper-geometric distribution and Kyoto Encyclopedia of Genes and Genomes (KEGG) enrichment analysis of cell pathways with FDR ≤ 0.05.

### Bioinformatics analysis

The expression of GPR137 [log_2_(TPM + 1)] and RAB8A [log_2_(TPM + 1)] in ovarian cancer (OV, T, *n* = 426) tissues and normal ovarian tissues (OV, N, *n* = 88) was assessed using the OV dataset in accordance with GEPIA (Gene Expression Profiling Interactive Analysis) database (http://gepia.cancer-pku.cn). In addition, GEPIA online tool was used to produce Kaplan–Meier survival plots (Disease Free Survival) with median in group cutoff (high-50% and low-50%) and 95% confidence interval, and the correlations between OV patient *GPR137* and *RAB8A* expression, *GPR137* and *PTCH1* expression as well as *RAB8A* and *PTCH1* expression with auto best cutoff.

### Statistics analyses

The results are shown as mean ± SDs. The GraphPad Prism 5.0 (GraphPad Software, Inc., San Diego, CA, USA) and Excel were used for statistical analysis. Statistical significance of the data was analyzed by unpaired Student’s t-test between two groups or with one-way ANOVA among multiple groups, which was assessed at *p* < 0.05 (* or #) and *p* < 0.01 (** or ##). Experiments were independently triplicated and representative experiments are shown.

## Results

### GPR137 is highly expressed in human OC and promotes OC cell propagation

**Fig. 1:**
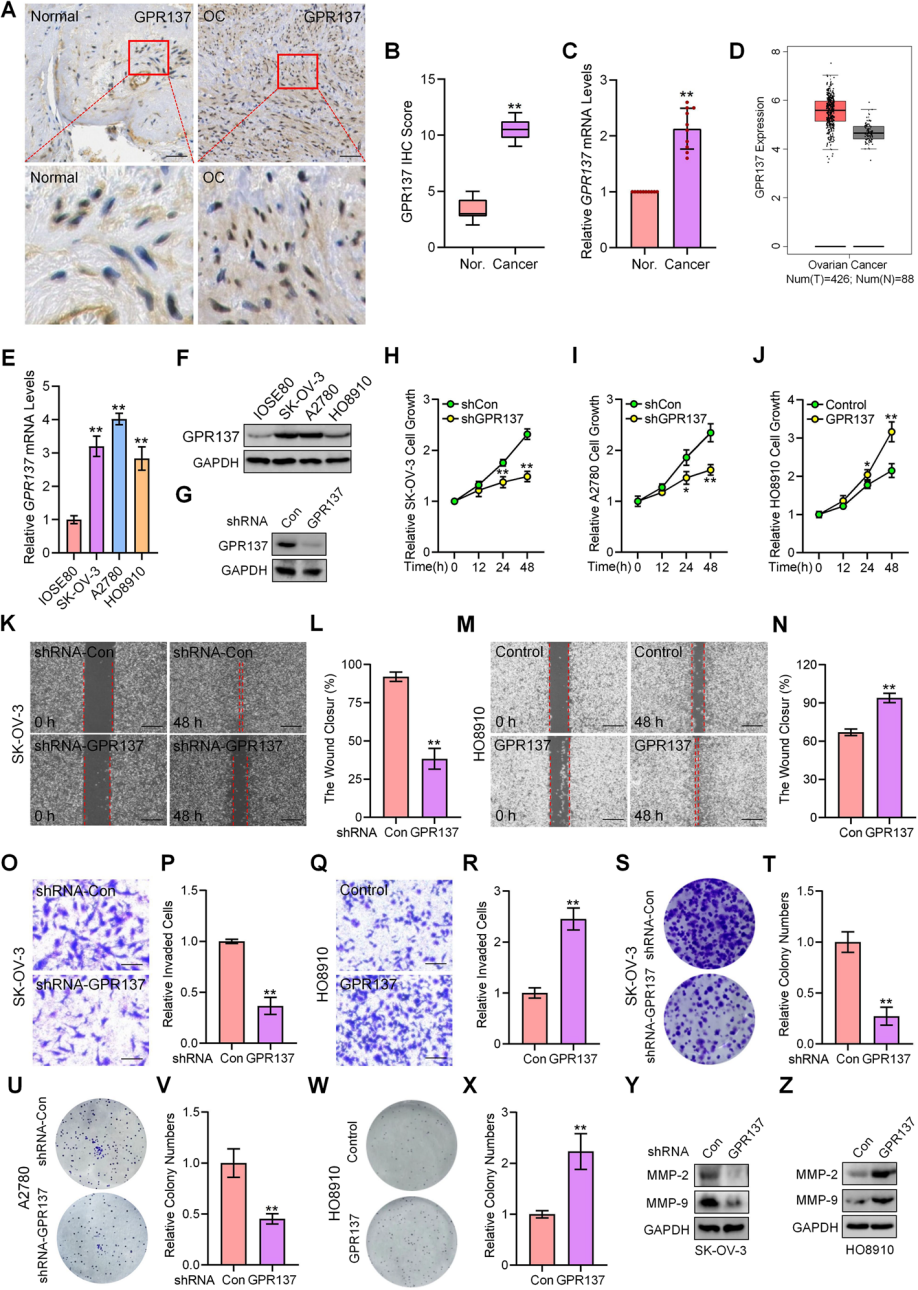
GPR137 is highly expressed in human OC tissues and promotes OC cell malignancy. **A** IHC staining for GPR137 by using paraffin-embedded sections of cancer tissues and adjacent normal gastric epithelial tissues from patients with OC. *N* = 10; bar, 20 μm. **B** IHC score for GPR137. *N* = 10. **C** The mRNA expression of *GPR137* in OC tissues and adjacent normal ovarian epithelial tissues. **D** Expression of GPR137 in OC tissues (T) and control groups (N) was revealed by GEPIA database. **E** The mRNA expression of *GPR137* in human normal ovarian epithelial IOSE80 cells, and human OC cell lines including SK-OV-3, A2780, and HO8910. **F** The protein expression of GPR137 in human normal ovarian epithelial IOSE80 cells, and human OC cell lines including SK-OV-3, A2780, and HO8910. **G** The protein expression of GPR137 in SK-OV-3 cells transfected with GPR137 shRNA or scrambled shRNA (Con). **H** CCK-8 assays of SK-OV-3 cells transfected with GPR137 shRNA (shGPR137) or scrambled shRNA (shCon) and cultured for the indicated time periods. **I** CCK-8 assays of A2780 cells transfected with GPR137 shRNA (shGPR137) or scrambled shRNA (shCon) and cultured for the indicated time periods. **J** CCK-8 assays of HO8910 cells transfected with a GPR137-expressing vector (GPR137) or a control empty vector (Control) and cultured for the indicated time periods. **K** Wound healing assays of SK-OV-3 cells transfected with GPR137 shRNA or scrambled shRNA (shRNA-Con) at 48 h. Bar, 100 μm. **L** Statistical analysis of unoccupied area in (**K**). **M** Wound healing assays of HO8910 cells transfected with a GPR137-expressing vector (GPR137) or a control empty vector (Control) at 48 h. Bar, 100 μm. **N** Statistical analysis of unoccupied area in (**M**). **O** Matrigel invasion assays of SK-OV-3 cells transfected with GPR137 shRNA or scrambled shRNA (shRNA-Con) for 24 h. Bar, 100 μm. **P** Quantitative analysis of (**O**). **Q** Matrigel invasion assays of HO8910 cells transfected with a GPR137-expressing vector (GPR137) or a control empty vector (Control) at 24 h. Bar, 100 μm. **R** Quantitative analysis of (**Q**). **S** Colony formation assays of SK-OV-3 cells infected with lentiviruses carrying GPR137 shRNA or scrambled shRNA (shRNA-Con). **T** Quantitative analysis of relative colony numbers in (S).**U** Colony formation assays of A2780 cells infected with lentiviruses carrying GPR137 shRNA or scrambled shRNA (shRNA-Con).**V** Quantitative analysis of relative colony numbers in (**U**). **W** Colony formation assays of HO8910 cells infected with lentiviruses expressing GPR137 or with control lentiviruses (Con). **X** Quantitative analysis of relative colony numbers in (**W**). **Y** The protein expression of MMP-2 and MMP-9 in SK-OV-3 cells transfected with GPR137 shRNA or scrambled shRNA (shRNA-Con). **Z** The protein expression of MMP-2 and MMP-9 in HO8910 cells transfected with a GPR137-expressing vector (GPR137) or a control empty vector (Con).*p < 0.05; **p < 0.01; error bar, SD

To investigate the role of GPR137 in OC, we first determined its clinical implication by comparing the expression of GPR137 in 10 pairs of OC tissues and adjacent non-tumor tissues. Both the GPR137 mRNA and protein expression levels were markedly upregulated in the obtained 10 pairs of OC tissues (Fig. 1A-C), which can be additionally verified in GEPIA database, showing the increased GPR137 expression in OC samples (T, Fig. 1D). Consistently, the mRNA and protein expression of GPR137 was obviously higher in human OC cell lines, including SK-OV-3, A2780, and HO8910, than that in human normal ovarian epithelial IOSE80 cells (Fig. 1E,F). The above data suggest that the aberrantly elevated expression of GPR137 is correlated with OC.

Given the relatively higher protein expression of GPR137 in SK-OV-3 and A2780 OC cells (Fig. 1F), we took advantage of these two cell lines for further loss-of-function experiments while performed gain-of-function experiments in HO8910 cells to investigate the biological function of GPR137 in OC. Knockdown of GPR137 with transfection of GPR137-shRNA that decreased GPR137 protein expression by almost 60% (Fig. 1G) significantly inhibited SK-OV-3 and A2780 OC cell proliferation (Fig. 1H,I), suppressed SK-OV-3 cell migration and invasion (Fig. 1K,L,O,P), and repressed colony-forming ability of SK-OV-3 and A2780 cells (Fig. 1S-V), accompanied by the down-regulated protein expression of MMP-2 and MMP-9 (Fig. 1Y), two critical molecules associated with OC cell mobility (migration and invasion) and OC metastasis [29, 30]. In contrast, overexpression of GPR137 significantly promoted HO8910 OC cell proliferation (Fig. 1J), potentiated migratory and invasive capacities (Fig. 1M,N,Q,R), enhanced colony-forming ability (Fig. 1W,X), and increased MMP-2 and MMP-9 protein expression (Fig. 1Z). Taken together, these results demonstrate that GPR137 plays a tumor-stimulative role in OC.

### RAB8A is positively correlated with GPR137 expression in human OC

**Fig. 2:**
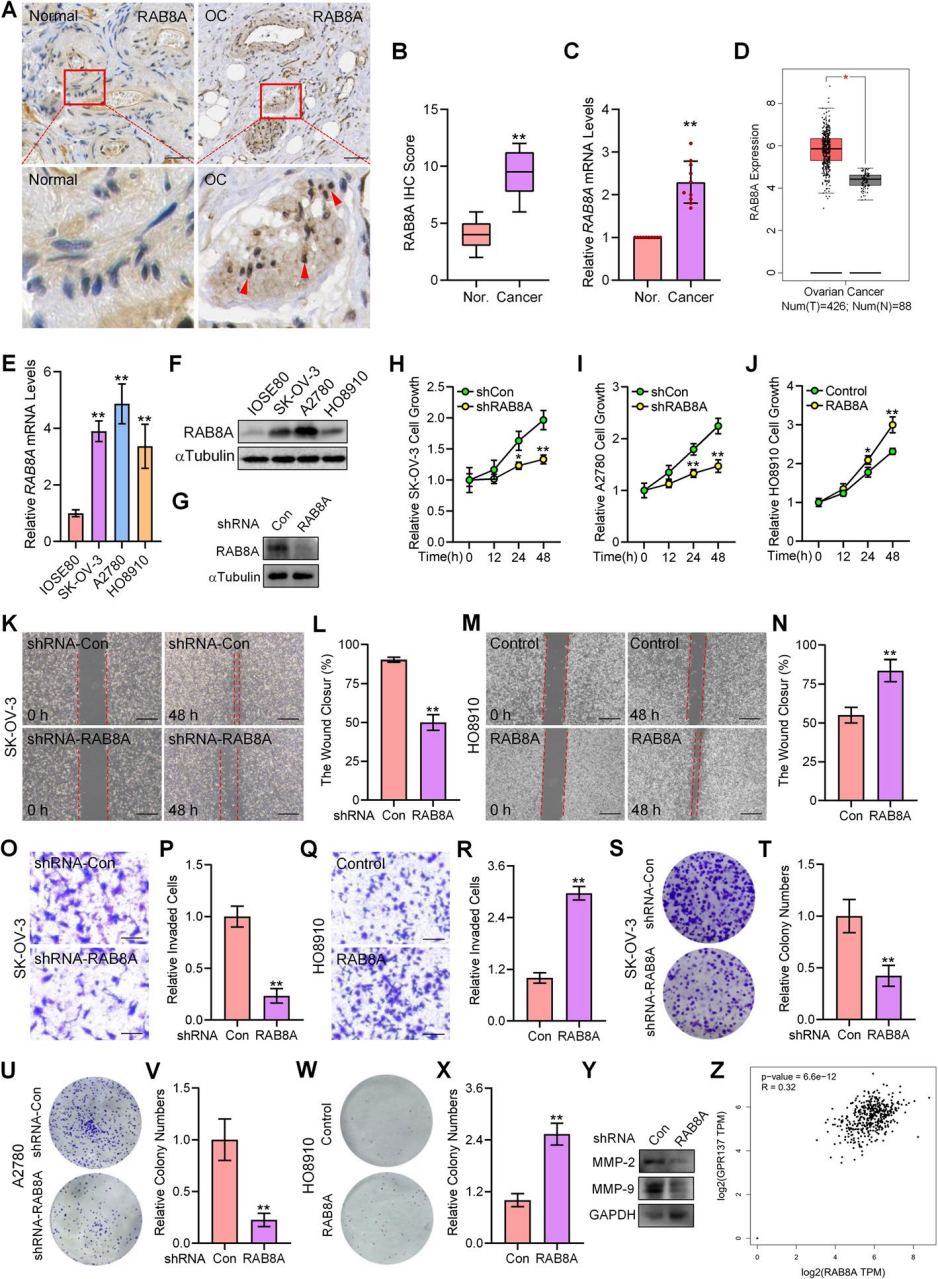
RAB8A is highly expressed in human OC tissues and promotes OC cell malignancy. **A** IHC staining for RAB8A by using paraffin-embedded sections of cancer tissues and adjacent normal gastric epithelial tissues from patients with OC. *N* = 10; bar, 20 μm. **B** IHC score for RAB8A. *N* = 10. **C** The mRNA expression of *RAB8A* in OC tissues and adjacent normal ovarian epithelial tissues. **D** Expression of RAB8A in OC tissues (T) and control groups (N) was revealed by GEPIA database. **E** The mRNA expression of *RAB8A* in human normal ovarian epithelial IOSE80 cells, and human OC cell lines including SK-OV-3, A2780, and HO8910. **F** The protein expression of RAB8A in human normal ovarian epithelial IOSE80 cells, and human OC cell lines including SK-OV-3, A2780, and HO8910. **G** The protein expression of RAB8A in SK-OV-3 cells transfected with RAB8A shRNA or scrambled shRNA (Con). **H** CCK-8 assays of SK-OV-3 cells transfected with RAB8A shRNA (shRAB8A) or scrambled shRNA (shCon) and cultured for the indicated time periods. **I** CCK-8 assays of A2780 cells transfected with RAB8A shRNA (shRAB8A) or scrambled shRNA (shCon) and cultured for the indicated time periods. **J** CCK-8 assays of HO8910 cells transfected with a RAB8A-expressing vector (RAB8A) or a control empty vector (Control) and cultured for the indicated time periods. **K** Wound healing assays of SK-OV-3 cells transfected with RAB8A shRNA or scrambled shRNA (shRNA-Con) at 48 h. Bar, 100 μm. **L** Statistical analysis of unoccupied area in (**K**). **M** Wound healing assays of HO8910 cells transfected with a RAB8A-expressing vector (RAB8A) or a control empty vector (Control) at 48 h. Bar, 100 μm. **N** Statistical analysis of unoccupied area in (**M**). **O** Matrigel invasion assays of SK-OV-3 cells transfected with RAB8A shRNA or scrambled shRNA (shRNA-Con) for 24 h. Bar, 100 μm. **P** Quantitative analysis of (**O**). **Q** Matrigel invasion assays of HO8910 cells transfected with a RAB8A-expressing vector (RAB8A) or a control empty vector (Control) at 24 h. Bar, 100 μm. **R** Quantitative analysis of (**Q**). **S** Colony formation assays of SK-OV-3 cells infected with lentiviruses carrying RAB8A shRNA or scrambled shRNA (shRNA-Con). **T** Quantitative analysis of relative colony numbers in (**S**). **U** Colony formation assays of A2780 cells infected with lentiviruses carrying RAB8A shRNA or scrambled shRNA (shRNA-Con). **V** Quantitative analysis of relative colony numbers in (**U**). **W** Colony formation assays of HO8910 cells infected with lentiviruses expressing RAB8A or with control lentiviruses (Con). **X** Quantitative analysis of relative colony numbers in (**W**). **Y** The protein expression of MMP-2 and MMP-9 in SK-OV-3 cells transfected with RAB8A shRNA or scrambled shRNA (shRNA-Con). **Z** GEPIA database displayed the correlation between *GPR137* and *RAB8A*. *p < 0.05; **p < 0.01; error bar, SD

As a member of Ras superfamily, RAB8 acts as a key regulator in intracellular membrane trafficking. Previous studies have demonstrated that RAB8 plays important roles in cancer cell migration, polarization, and signal transduction [31]. RAB8A is one major subtype of RAB8 and is reported to be involved in female reproductive carcinogenesis such as cervical cancer and endometrial cancer [32, 33], indicating its role as a potential gynecological tumor biomarker. Similar to GPR137 expression pattern, RAB8A expression was significantly up-regulated in the 10 pairs of OC tissue samples in comparison with control normal ovarian epithelial tissues, as determined by IHC staining and mRNA level examination (Fig. 2A-C), which was in accordance with the data from GEPIA database (Fig. 2D). As expected, the mRNA and protein expression of RAB8A was apparently higher in all the three human OC cell lines than that in human normal ovarian epithelial cells (Fig. 2E,F), indicating the aberrantly up-regulated expression of RAB8A could be correlated with OC progression.

**Fig. 3:**
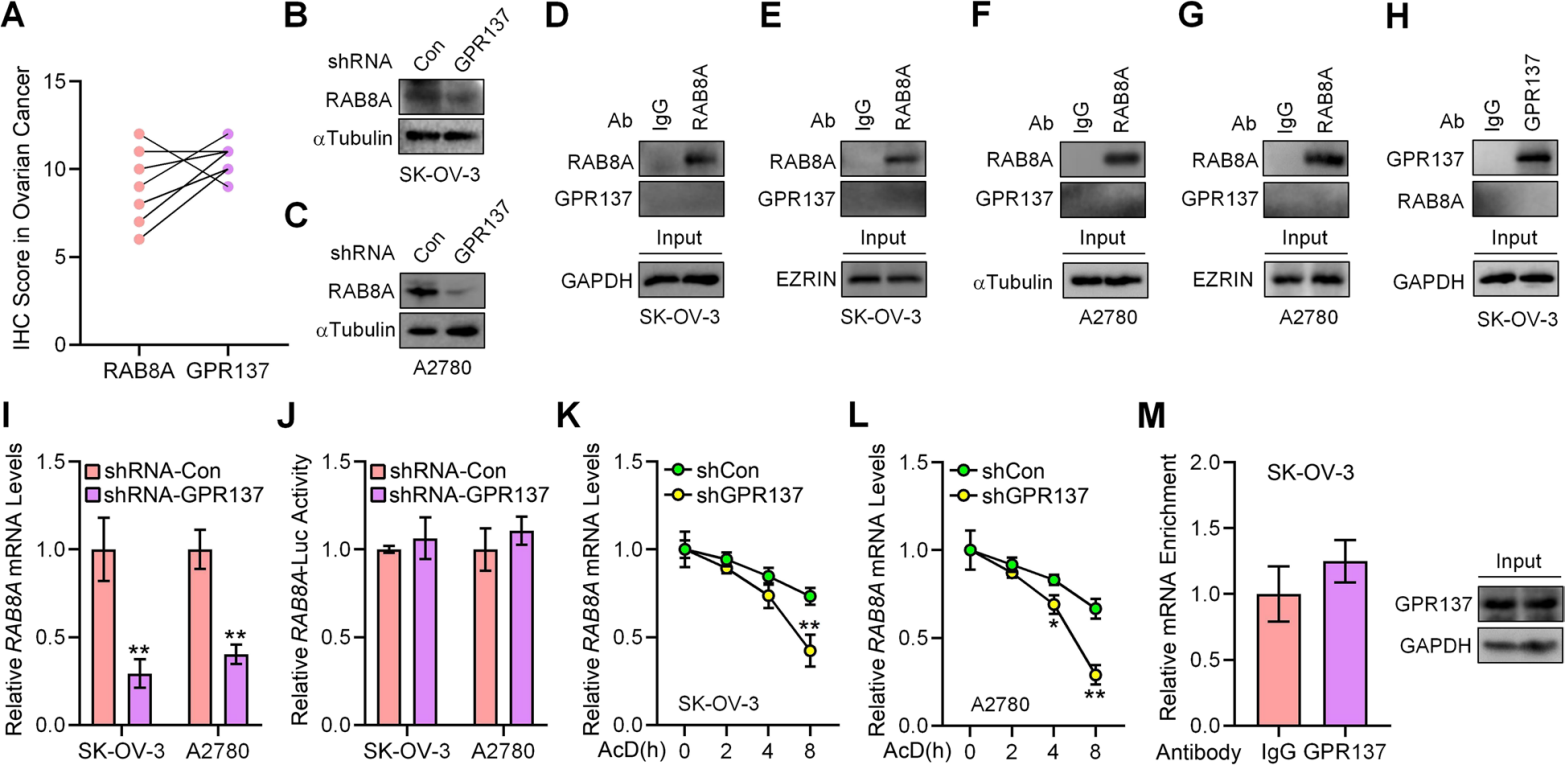
GPR137 enhances *RAB8A* mRNA stability. **A** IHC score for GPR137 and RAB8A in OC tissues. **B** The protein expression of RAB8A in SK-OV-3 cells transfected with GPR137 shRNA or scrambled shRNA (Con). **C** The protein expression of RAB8A in A2780 cells transfected with GPR137 shRNA or scrambled shRNA (Con). **D** Co-immunoprecipitation of endogenous GPR137 and RAB8A in SK-OV-3 cells. IP: RAB8A; WB: GPR137. IgG was used as a negative control. **E** Co-immunoprecipitation of endogenous GPR137 and RAB8A in separated membrane fraction from SK-OV-3 cells. IP: RAB8A; WB: GPR137. IgG was used as a negative control and EZRIN was used as loading control for membrane protein. **F** Co-immunoprecipitation of endogenous GPR137 and RAB8A in A2780 cells. IP: RAB8A; WB: GPR137. IgG was used as a negative control. **G** Co-immunoprecipitation of endogenous GPR137 and RAB8A in separated membrane fraction from A2780 cells. IP: RAB8A; WB: GPR137. IgG was used as a negative control and EZRIN was used as loading control for membrane protein. **H** Co-immunoprecipitation of endogenous GPR137 and RAB8A in SK-OV-3 cells. IP: GPR137; WB: RAB8A. IgG was used as a negative control. **I** The mRNA expression of *RAB8A* in SK-OV-3 and A2780 cells transfected with GPR137 shRNA or scrambled shRNA (shRNA-Con). **J** The *RAB8A* promoter-luciferase (*RAB8A*-Luc) activities in SK-OV-3 and A2780 cells transfected with GPR137 shRNA or scrambled shRNA (shRNA-Con). **K** The mRNA levels of *RAB8A* in SK-OV-3 cells transfected with GPR137 shRNA (shGPR137) or scrambled shRNA (shCon) and cultured with actinomycin D (AcD) for the indicated time periods. **L** The mRNA levels of *RAB8A* in A2780 cells transfected with GPR137 shRNA (shGPR137) or scrambled shRNA (shCon) and cultured with actinomycin D (AcD) for the indicated time periods. **M** qRT-PCR analysis of co-precipitated *RAB8A* mRNA by GPR137 antibody in the RIP assay in SK-OV-3 cells. IgG was used as a negative control. Protein loading was also determined (Input). *p < 0.05; **p < 0.01; error bar, SD

Given the mRNA and protein amount of endogenous RAB8A in three different OC cell lines (Fig. 2E,F), we subsequently carried out experiments of RAB8A biological function by silencing RAB8A in SK-OV-3 and A2780 cells while overexpressing RAB8A in HO8910 cells. Suppression of RAB8A expression by RAB8A-shRNA significantly declined SK-OV-3 and A2780 OC cell proliferation (Fig. 2G-I), inhibited migration and invasion in SK-OV-3 cells (Fig. 2K,L,O,P), decreased colony numbers of SK-OV-3 and A2780 cells (Fig. 2S-V), and reduced protein expression of MMP-2 and MMP-9 (Fig. 2Y). Conversely, overexpression of RAB8A significantly induced HO8910 cells proliferation (Fig. 2J), promoted migratory and invasive capacities (Fig. 2M,N,Q,R), and increased colony numbers (Fig. 2W,X). Collectively, the above results illustrate that RAB8A plays an important role in OC. Given the similar effects of RAB8A and GPR137 on OC cell biological function, we thereby inquired if a correlation between the two proteins exists in OC tissues. Indeed, we found a positive correlation between the expression of GPR137 and RAB8A using GEPIA database (Fig. 2Z), which was additionally verified by scoring and analyzing the IHC staining of GPR137 and RAB8A in collected clinical OC tissues (Fig. 3A), reinforcing the notion that GPR137 and RAB8A participate in OC progression.

### GPR137 increases RAB8A expression by promoting RAB8A mRNA stability

Given the similar protein expression pattern in OC (Fig. 3A), we postulated the possible regulation between RAB8A and GPR137. As expected, knockdown of GPR137 suppressed RAB8A protein expression in both SK-OV-3 and A2780 cells (Fig. 3B,C), although alteration of RAB8A expression by RAB8A-shRNA in SK-OV-3 cells or RAB8A-expressing vector in HO8910 cells did not show apparent effects on GPR137 expression (Figure S1A,B). While GPR137 and RAB8A are distributed in both cellular cytoplasm and membrane [34, 35]. GPR137 failed to interact with RAB8A in either cytoplasm or separated OC cell membrane, which was determined by membrane-cytoplasmic separation assay-followed reciprocal co-IP assays (Fig. 3D-H). Intriguingly, silencing of GPR137 resulted in significant reduction of *RAB8A* mRNA (Fig. 3I), while unexpectedly, there was no significant change in *RAB8A* promoter-luciferase reporter assays (*RAB8A*-Luc) (Fig. 3J), suggesting GPR137 in not involved in regulating *RAB8A* transcription.

To gain insight into the mechanisms that GPR137 regulates *RAB8A* mRNA levels, we next performed an RNA stability assay and examined the effect of GPR137 on *RAB8A* mRNA using actinomycin D (AcD). As a result, a significant decrease in *RAB8A* mRNA was observed in both SK-OV-3 and A2780 cells expressing GPR137-shRNA (shGPR137) after AcD treatment for 4 h and 8 h (Fig. 3K,L), with a reduction of almost 55% (SK-OV-3, 8 h) and 75% (A2780, 8 h), respectively, although the endogenous *RAB8A* transcripts were not associated with GPR137 protein (Fig. 3M). In summary, these data demonstrate that GPR137 elevates RAB8A expression by increasing *RAB8A* mRNA stability.

### Identification of HH signaling as the downstream of the GPR137-RAB8A axis

**Fig. 4:**
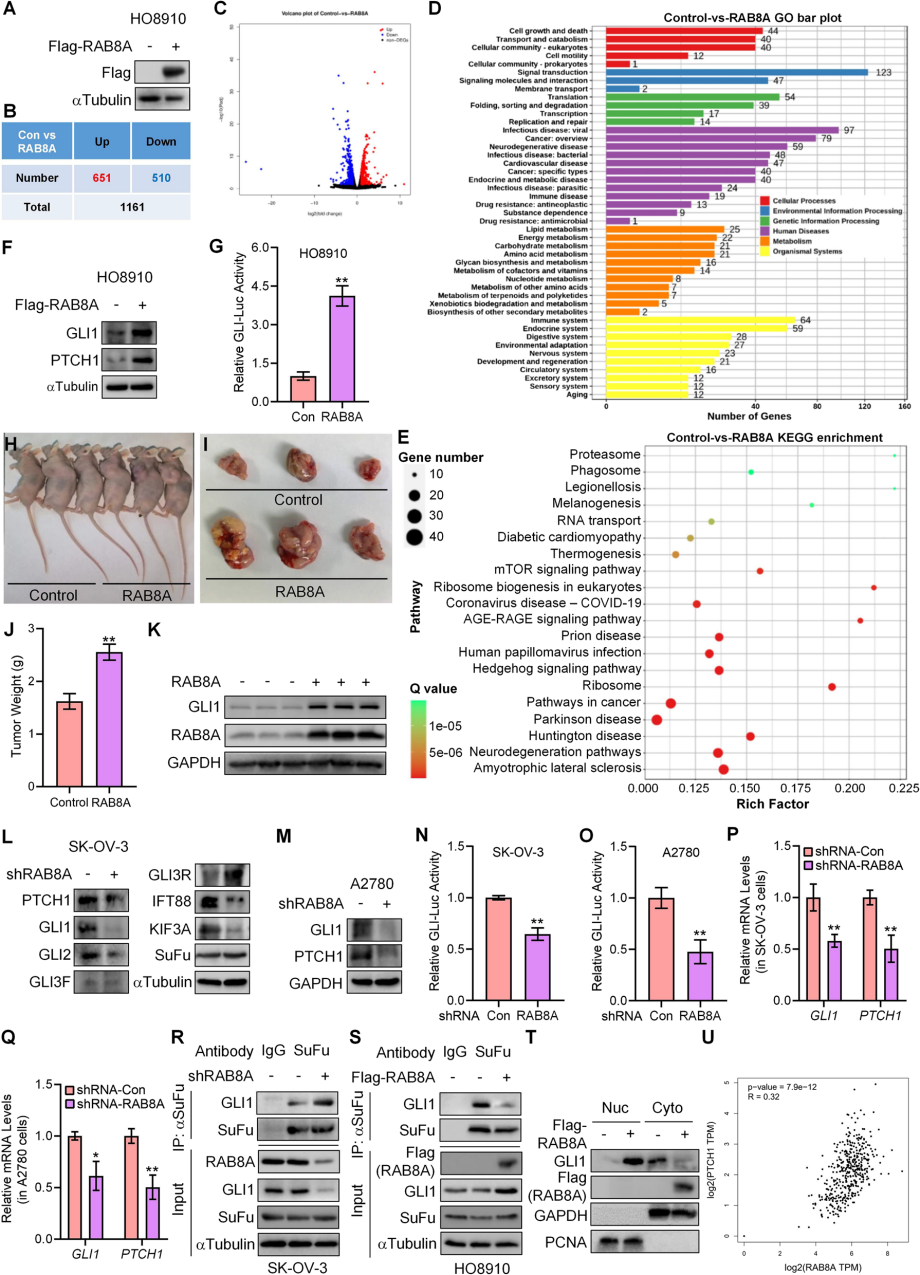
Identification of HH signaling as the downstream of RAB8A. **A** The protein levels of exogenous RAB8A (Flag) in RAB8A cells transfected with a Flag-tagged RAB8A (Flag-RAB8A, +) or an empty vector (Flag-RAB8A, -) for 24 h were detected by western blot. **B** The number of genes whose expression was altered by Flag-RAB8A-expression is summarized in the table. **C** Volcano scatter plot for the genes in (B). **D** GO analysis was conducted to compare the gene expression from RAB8A group and the control. **E** KEGG pathway enrichment analysis was conducted to compare the gene expression from RAB8A group and the control. **F** The protein expression of GLI1 and PTCH1 in HO8910 cells transfected with RAB8A (Flag-RAB8A, +) or an empty vector (Flag-RAB8A, -). **G** GLI luciferase (GLI-Luc) activities in HO8910 cells transfected with RAB8A or an empty vector (Con). **H** Nude mice with xenografts derived from HO8910 cells stably expressing RAB8A or control viruses. **I** Image showing the size of the representative tumor xenografts from two groups. **J** Weights of xenografts derived from HO8910 cells stably expressing RAB8A or control viruses at 21 d post inoculation. **K** The protein levels of GLI1 and RAB8A in tumors from HO8910 cells stably expressing RAB8A or control viruses. **L** The protein expression of PTCH1, GLI1, GLI2, GLI3-full length (GLI3F), GLI3-repressor (GLI3R), IFT88, KIF3A, and SuFu in SK-OV-3 cells transfected with RAB8A shRNA (shRAB8A, +) or scrambled shRNA (shRAB8A, -). **M** The protein expression of GLI1 and PTCH1 in A2780 cells transfected with RAB8A shRNA (shRAB8A, +) or scrambled shRNA (shRAB8A, -). **N** GLI luciferase (GLI-Luc) activities in SK-OV-3 cells transfected with RAB8A shRNA (shRNA RAB8A) or scrambled shRNA (shRNA Con). **O** GLI luciferase (GLI-Luc) activities in A2780 cells transfected with RAB8A shRNA (shRNA RAB8A) or scrambled shRNA (shRNA Con). **P** The mRNA levels of *GLI1* and *PTCH1* in SK-OV-3 cells transfected with RAB8A shRNA (shRNA-RAB8A) or scrambled shRNA (shRNA-Con). **Q** The mRNA levels of *GLI1* and *PTCH1* in A2780 cells transfected with RAB8A shRNA (shRNA-RAB8A) or scrambled shRNA (shRNA-Con). **R** Co-immunoprecipitation of endogenous SuFu and GLI1 in the presence of RAB8A shRNA (shRAB8A) in SK-OV-3 cells. IP: SuFu; WB: GLI1. IgG was used as a negative control. **S** Co-immunoprecipitation of endogenous SuFu and GLI1 in the presence of ectopic Flag-RAB8A in HO8910 cells. IP: SuFu; WB: GLI1. IgG was used as a negative control. **T** Nucleo-cytoplasmic separation assays in HO8910 cells transfected with Flag-RAB8A or an empty vector (Flag-RAB8A, -), and protein levels of GLI1 were detected by western blot. **U** GEPIA database displayed the correlation between *RAB8A* and *PTCH1*. *p < 0.05; **p < 0.01; error bar, SD

We were next eager to clarify the molecular mechanisms underlying the function of RAB8A in OC. To this end, an RNA sequencing (RNA-Seq) assay using HO8910 cells transfected with a RAB8A-expressing vector (Flag-RAB8A) or a control empty vector was applied, and the transfection efficiency was determined by western blot (Fig. 4A). Totally, 1161 transcripts with log_2_(Fold Change) > 1 or < -1 were significantly changed upon RAB8A-overexpression, among which 651 were up-regulated and 510 were down-regulated (Fig. 4B,C), respectively, suggesting that variation of RAB8A expression markedly altered the transcriptome. Bioinformatics results of GO and KEGG analysis revealed that the exogenous expression of RAB8A exerted effects on many important processes in cell biology and environmental processing, such as cell growth and cell motility, signaling transduction, and signaling molecules and interaction, as well as human diseases such as cancer (Fig. 4D,E). Consistently, the enrichment analysis of the top 20 pathways from KEGG also exhibited that RAB8A was correlated with multiple pathways in cancer, including the Hedgehog (HH) signaling pathway (Fig. 4E), based on which and in combination with the heat-map data showing marked increase of GLI1 expression (Figure S2A), a target in HH signaling, we finally selected the HH signaling as a potential candidate.

As expected, overexpression of RAB8A significantly induced the HH signaling activity, including the upregulated protein expression of HH target GLI1 and PTCH1 as well as the GLI-Luc activities (Fig. 4F,G). Consistently, compared with the HO8910 cells infected with control viruses, the RAB8A-overexpressing lentiviruses-infected HO8910 cells resulted in the xenografts with the significantly increased tumor weight and tumor volume in nude mice (Fig. 4H-J), and the protein expression of GLI1 was markedly induced in corresponding tumor tissues where RAB8A was ectopicly expressed (Fig. 4K). On the contrary, inhibition of RAB8A deactivated HH activity in both SK-OV-3 and A2780 cells, showing decreased mRNA and protein expression of GLI1 and PTCH1 and reduced GLI-Luc activities (Fig. 4L-Q). Given that knockdown of RAB8A also affected the expression pattern of key components in HH pathway, such as GLI2, GLI3F/R, KIF3A and IFT88 [18] (Fig. 4L), we hypothesized that RAB8A might mediate HH deactivation through SuFu, a main repressor in HH pathway that ties up GLI1 and therefore impedes GLI1 nuclear-translocation [17]. Indeed, silencing of RAB8A in SK-OV-3 cells effectively increased the binding of SuFu with GLI1 (Fig. 4R), whereas overexpression of RAB8A obviously induced the dissociation of SuFu from its binding with GLI1 (Fig. 4S), resulting in translocation of GLI1 from cytoplasm into the nucleus (Fig. 4T). In addition, a positive correlation between the expression of *RAB8A* and the HH target *PTCH1* was found in GEPIA database (Fig. 4U), also providing with evidence that RAB8A contributes to OC progression through activating HH signaling.

### GPR137-RAB8A promotes OC progression by activating HH signaling

**Fig. 5:**
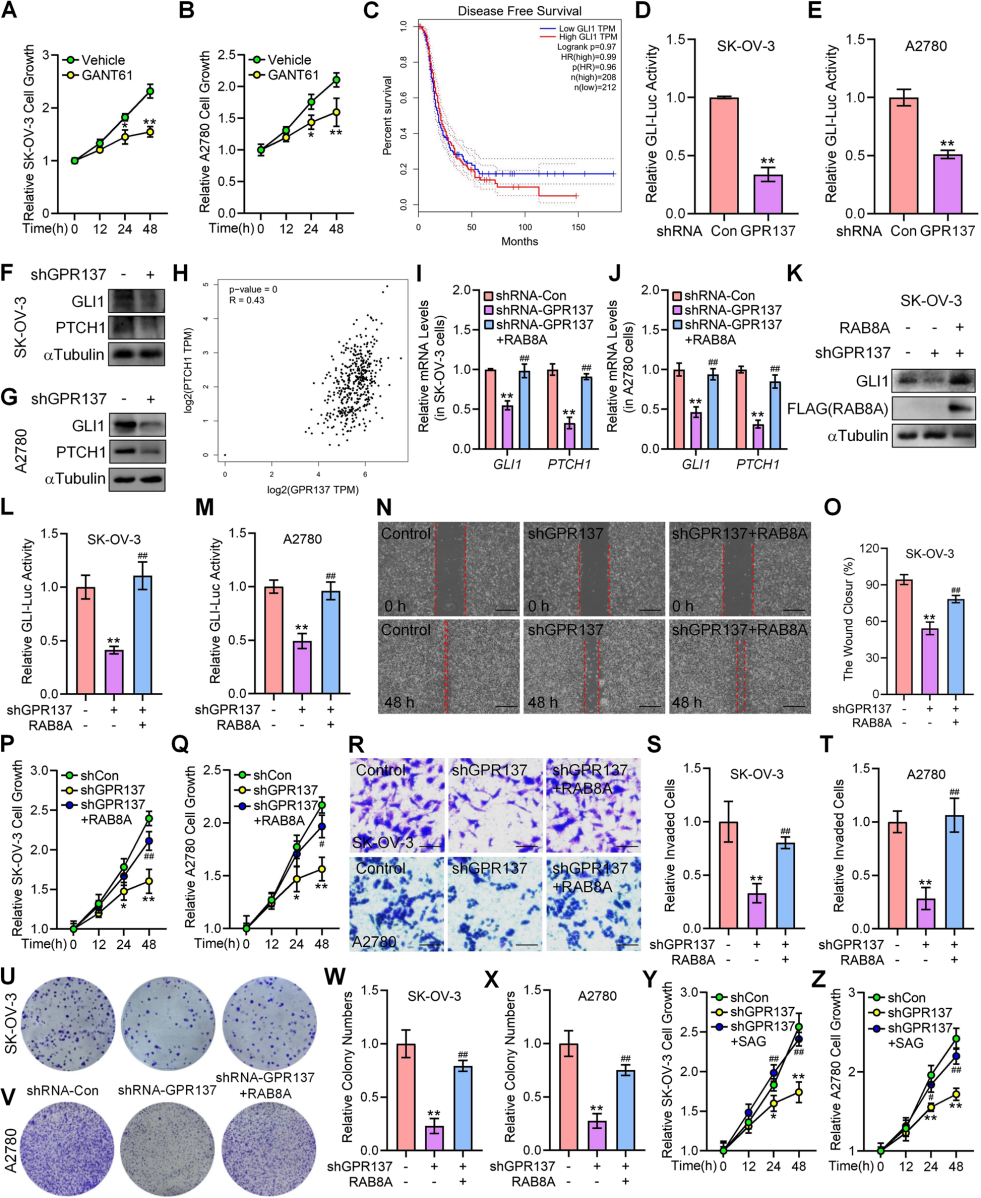
GPR137-RAB8A promotes OC progression through HH signaling. **A** CCK-8 assays of SK-OV-3 cells treated with GANT61 or vehicle and cultured for the indicated time periods. **B** CCK-8 assays of A2780 cells treated with GANT61 or vehicle and cultured for the indicated time periods. **C** Kaplan–Meier analysis of disease free survival for OC patients with altered expression of GLI1. **D** GLI luciferase (GLI-Luc) activities in SK-OV-3 cells transfected with GPR137 shRNA (shRNA GPR137) or scrambled shRNA (shRNA Con). **E** GLI luciferase (GLI-Luc) activities in A2780 cells transfected with GPR137 shRNA (shRNA GPR137) or scrambled shRNA (shRNA Con). **F** The protein expression of GLI1 and PTCH1 in SK-OV-3 cells transfected with GPR137 shRNA (shGPR137, +) or scrambled shRNA (shGPR137, -). **G** The protein expression of GLI1 and PTCH1 in A2780 cells transfected with GPR137 shRNA (shGPR137, +) or scrambled shRNA (shGPR137, -). **H** GEPIA database displayed the correlation between *GPR137* and *PTCH1*. **I** The mRNA levels of *GLI1* and *PTCH1* in SK-OV-3 cells transfected with GPR137 shRNA (shRNA-GPR137) or scrambled shRNA (shRNA-Con) in combination with a RAB8A-expressing vector (RAB8A). **J** The mRNA levels of *GLI1* and *PTCH1* in A2780 cells transfected with GPR137 shRNA (shRNA-GPR137) or scrambled shRNA (shRNA-Con) in combination with a RAB8A-expressing vector (RAB8A). **K** The protein levels of GLI1 in SK-OV-3 cells transfected with GPR137 shRNA (shGPR137, +) or scrambled shRNA (shGPR137, -) in combination with a RAB8A-expressing vector (RAB8A,—or +). **L** GLI luciferase (GLI-Luc) activities in SK-OV-3 cells transfected with GPR137 shRNA (shGPR137, +) or scrambled shRNA (shGPR137, -) in combination with a RAB8A-expressing vector (RAB8A,—or +). **M** GLI luciferase (GLI-Luc) activities in A2780 cells transfected with GPR137 shRNA (shGPR137, +) or scrambled shRNA (shGPR137, -) in combination with a RAB8A-expressing vector (RAB8A,—or +). **N** Wound healing assays of SK-OV-3 cells transfected with GPR137 shRNA (shGPR137) or scrambled shRNA (Control) in combination with a RAB8A-expressing vector (RAB8A,—or +) at 48 h. Bar, 100 μm. **O** Statistical analysis of unoccupied area in (**N**). **P** CCK-8 assays of SK-OV-3 cells transfected with GPR137 shRNA (shGPR137) or scrambled shRNA (shCon) in combination with a RAB8A-expressing vector (RAB8A,—or +) and cultured for the indicated time periods. **Q** CCK-8 assays of A2780 cells transfected with GPR137 shRNA (shGPR137) or scrambled shRNA (shCon) in combination with a RAB8A-expressing vector (RAB8A,—or +) and cultured for the indicated time periods. **R** Matrigel invasion assays of SK-OV-3 (upper) and A2780 (lower) cells transfected with GPR137 shRNA (shGPR137) or scrambled shRNA (Control) in combination with a RAB8A-expressing vector (RAB8A,—or +) for 24 h. Bar, 100 μm. **S** Quantitative analysis of (R, upper: SK-OV-3). **T** Quantitative analysis of (R, lower: A2780). **U** Colony formation assays of SK-OV-3 cells infected with lentiviruses carrying GPR137 shRNA or scrambled shRNA (shRNA-Con) in combination with the RAB8A-expressing lentiviruses or control lentiviruses (RAB8A,—or +). **V** Colony formation assays of A2780 cells infected with lentiviruses carrying GPR137 shRNA or scrambled shRNA (shRNA-Con) in combination with the RAB8A-expressing lentiviruses or control lentiviruses (RAB8A,—or +). **W** Quantitative analysis of relative colony numbers in (**U**). **X** Quantitative analysis of relative colony numbers in (**V**). **Y** CCK-8 assays of SK-OV-3 cells transfected with GPR137 shRNA (shGPR137) or scrambled shRNA (shCon) in combination with SAG-treatment or vehicle and cultured for the indicated time periods. **Z** CCK-8 assays of A2780 cells transfected with GPR137 shRNA (shGPR137) or scrambled shRNA (shCon) in combination with SAG-treatment or vehicle and cultured for the indicated time periods. *, #*p* < 0.05; **, ##*p* < 0.01; error bar, SD

Inhibition of HH signaling by specific GLI1 antagonist GANT61 [36] significantly reduced OC cells proliferation (Fig. 5A,B), particularly at 24 h and 48 h, and consistently, upregulation of GLI1 expression was significantly associated with lower diseases-free survival (DFS) of OC patients (Fig. 5C), indicating aberrant activation of HH signaling strongly correlates with poor outcomes of OC. Similar to RAB8A, knockdown of GPR137 in SK-OV-3 and A2780 cells significantly decreased the GLI-Luc activities (Fig. 5D,E) and inhibited the protein expression of GLI1 and PTCH1 (Fig. 5F,G), whereas overexpression of RAB8A increased the protein expression of GLI1 and PTCH1 in HO8910 cells (Figure S3A). Consistently, GEPIA database showed that the expression of *GPR137* was positively correlated with the HH target *PTCH1* expression (Fig. 5H), demonstrating the positive effect of GPR137 on HH signaling transduction.

To explore whether GPR137 modulates HH signaling through RAB8A, we next performed experiments by inhibiting GPR137 expression and simultaneously upregulating RAB8A expression in OC cells. Overexpression of RAB8A effectively restored the GPR137 silencing-derived negative effects on the expression of GLI1 and PTCH1 (Fig. 5I-K), and the GLI-Luc activities in both SK-OV-3 and A2780 OC cells (Fig. 5L,M), as well as the SK-OV-3 cell migratory capacity (Fig. 5N,O), the proliferation, invasion and colony numbers in SK-OV-3 and A2780 OC cells (Fig. 5P-X). Similarly, activation of HH signaling by SAG, a specific agonist against receptor Smoothened (SMO) [17], significantly attenuated the GPR137 shRNA-suppressed proliferation, invasion, and colony formation capacity in SK-OV-3 and A2780 OC cells (Fig. 5Y,Z, Figure S4A-G).

Consistently, administration of GDC-0449 almost completely diminished the GPR137-trigged proliferation, invasion, and colony formation in HO89010 cells (Figure S4H-L). Similarly, silencing of RAB8A not only effectively restored the GPR137-induced HH activity including increased GLI-Luc activities and upregulated RNA expression of *GLI1* and *PTCH1* (Figure S4M,N), but also obviously weakened the GPR137 overexpression-promoted proliferation, invasion, and colony formation in HO89010 cells (Figure S4O-S), demonstrating that GPR137-RAB8A signal participates in OC propagation through activating HH signaling.

### HH signaling activation reciprocally activates GPR137-RAB8A

**Fig. 6:**
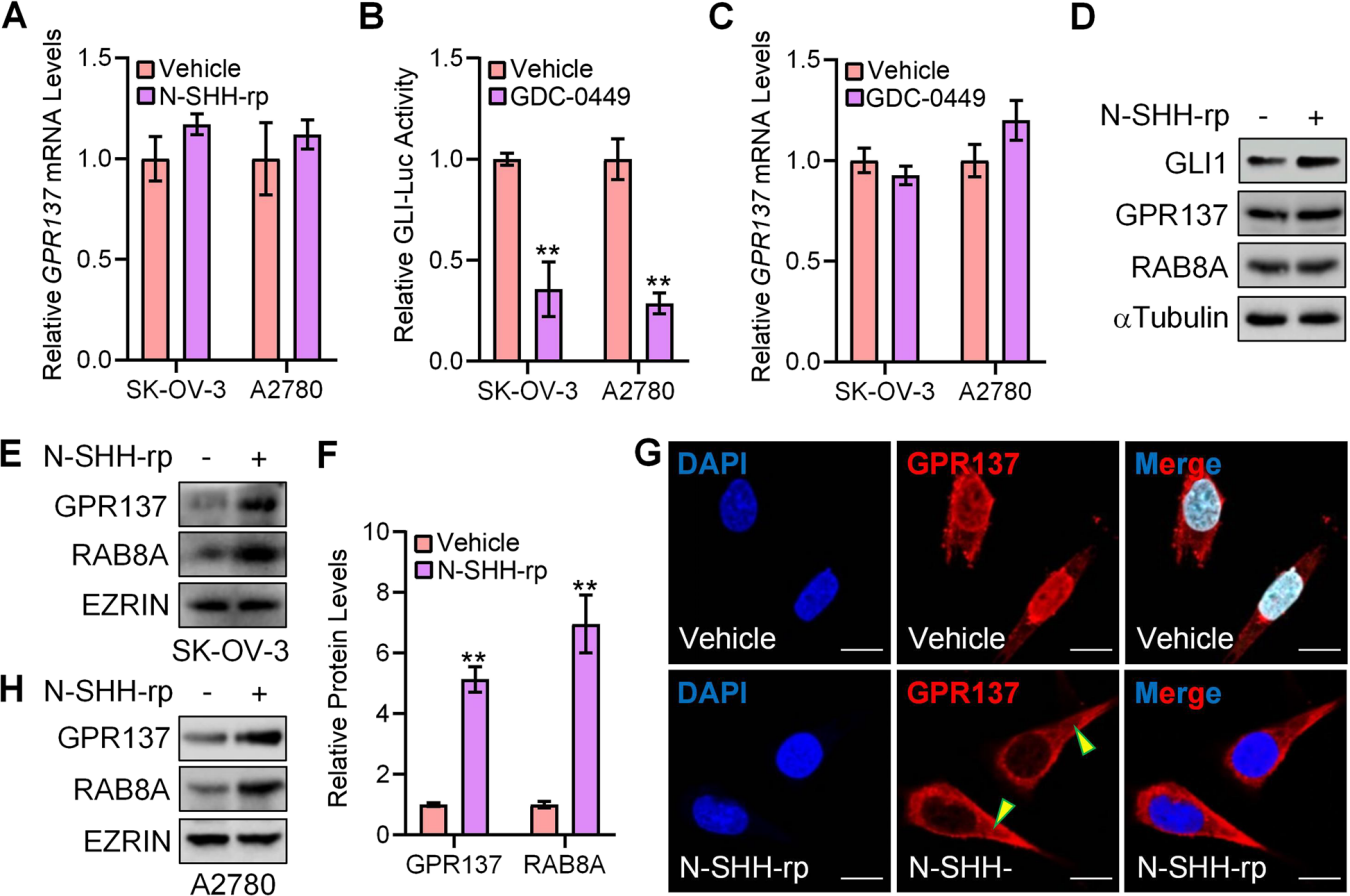
HH signaling activates GPR137 and RAB8A. **A** The mRNA levels of *GPR137* in SK-OV-3 and A2780 cells treated with N-terminus of human SHH recombinant protein (N-SHH-rp) or vehicle and cultured for 24 h. **B** GLI luciferase (GLI-Luc) activities in SK-OV-3 and A2780 cells treated with GDC-0449 or vehicle and cultured for 24 h. **C** The mRNA levels of *GPR137* in SK-OV-3 and A2780 cells treated with GDC-0449 or vehicle and cultured for 24 h. **D** The protein levels of GLI1, GPR137, and RAB8A in SK-OV-3 cells treated with N-SHH-rp (N-SHH-rp, +) or vehicle (N-SHH-rp, -) and cultured for 24 h. **E** The protein levels of GPR137 and RAB8A in separated membrane fraction from SK-OV-3 cells treated with N-SHH-rp (N-SHH-rp, +) or vehicle (N-SHH-rp, -) and cultured for 24 h. EZRIN was used as loading control for membrane protein. **F** Quantification via densitometry and statistical analysis of bands of GPR137 and RAB8A in (E), respectively. **G** Immunofluorescent staining for GPR137-derived signal in SK-OV-3 cells treated with N-SHH-rp or vehicle and cultured for 24 h. Nuclei were stained with DAPI. Bar,5 μm. **H** The protein levels of GPR137 and RAB8A in separated membrane fraction from A2780 cells treated with N-SHH-rp (N-SHH-rp, +) or vehicle (N-SHH-rp, -) and cultured for 24 h. EZRIN was used as loading control for membrane protein.** *p* < 0.01; error bar, SD

To investigate the possible regulation of GPR137 and/or RAB8A by HH, we next treated OC cells with either human N-SHH recombinant protein (N-SHH-rp) or GDC-0449, an HH signaling antagonist [17], and detected the expression of GPR137 and RAB8A. While N-SHH-rp administration induced GLI1 protein expression but GDC-0449 significantly deactivated HH signaling with the decreased GLI-Luc activities in SK-OV-3 and A2780 OC cells, neither N-SHH-rp nor GDC-0449 affected either the RNA expression or protein expression of GPR137 and RAB8A, unexpectedly (Fig. 6A-D). However, as determined by membrane-cytoplasmic separation assay and IF staining in SK-OV-3 cells and A2780 cells, the membrane distribution of both GPR137 and RAB8A proteins was markedly increased by N-SHH-rp (Fig. 6E-H), indicating HH activation positively altered the activity of GPR137 and RAB8A, which formed a positive feedback loop for GPR137-RAB8A-HH axis regulation in OC carcinogenesis.

### Suppression of GPR137-RAB8A hampered OC progression in vivo by deactivating HH signaling

**Fig. 7:**
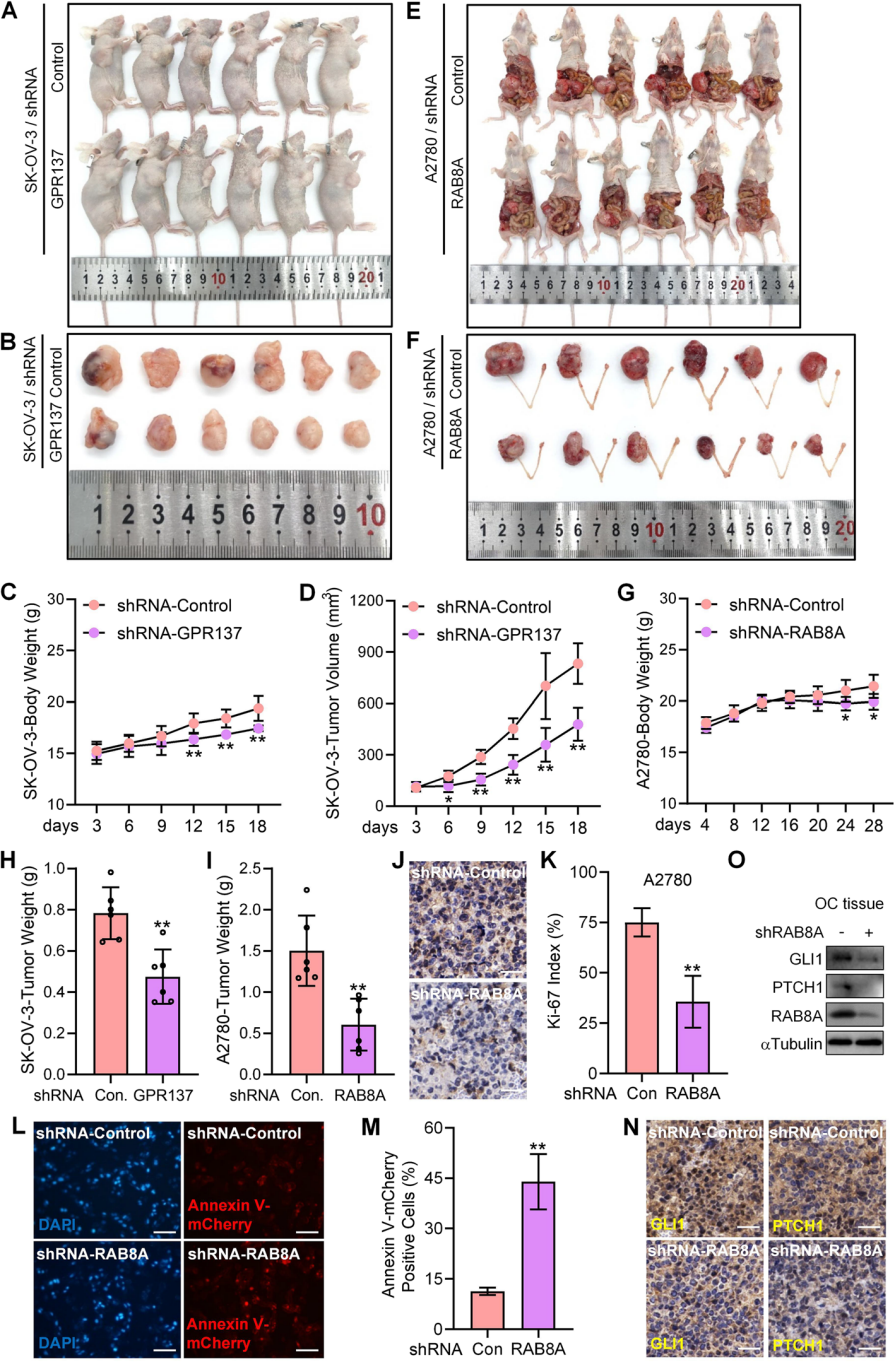
Inhibition of GPR137-RAB8A hampers OC progression in vivo. **A** Nude mice with xenografts derived from SK-OV-3 cells stably expressing GPR137-shRNA (shRNA-GPR137) or control viruses (shRNA-Control). *N* = 6. **B** Image showing the size of the tumor xenografts from two groups. **C** Body weight of mice in (**A**) was examined on day 3, 6, 9, 12, 15, and 18, after tumor cell infection. **D** Tumor volume in (**A**) was measured on day 3, 6, 9, 12, 15, and 18, after tumor cell infection. **E** Nude mice with in situ ovarian tumor from A2780 cells stably expressing RAB8A-shRNA (shRNA-RAB8A) or control viruses (shRNA-Control). *N* = 6. **F** Image showing the size of the tumors from two groups. **G** Body weight of mice in (**E**) was examined on day 4, 8, 12, 16, 20, 24, and 28, after tumor cell infection. **H** Weights of xenografts derived from SK-OV-3 cells stably expressing GPR137-shRNA (shRNA-GPR137) or control viruses (shRNA-Control) on day 18 post inoculation. **I** Weights of ovarian tumors derived from A2780 cells stably expressing RAB8A-shRNA (shRNA-RAB8A) or control viruses (shRNA-Control) on day 28 post inoculation. **J** Ki67 staining for tumor tissues derived from A2780 cells stably expressing RAB8A-shRNA (shRNA-RAB8A) or control viruses (shRNA-Control) on day 28 post inoculation. Bar,100 μm. **K** Statistical analysis of Ki67-index (%) from (**J**). **L** Annexin V-mCherry staining of tumor tissues derived from A2780 cells stably expressing RAB8A-shRNA (shRNA-RAB8A) or control viruses (shRNA-Control) on day 28 post inoculation. Nuclei were stained with DAPI. Bar,100 μm. **M** Statistical analysis of Annexin V-mCherry positive cells (%) from (L). **N** The IHC staining of GLI1 and PTCH1 in formed in situ ovarian tumors derived from A2780 cells stably expressing RAB8A-shRNA or control viruses (shRNA-Control). Bar,100 μm. **O** The protein levels of GLI1, PTCH1, and RAB8A in formed in situ ovarian tumor tissues derived from A2780 cells stably expressing RAB8A-shRNA (shRAB8A, +) or control viruses (shRAB8A, -). Bar,100 μm.** *p* < 0.01; error bar, SD

To finally test the oncogenic role of GPR137-RAB8A signal axis in OC progression in vivo, we established a SK-OV-3 cell line and an A2780 cell line that stably expressed GPR137-shRNA and RAB8A-shRNA by infection of levtiviruses carrying the shRNA sequences targeting *GPR137* or *RAB8A*, respectively. Compared with the corresponding control SK-OV-3 cells and A2780 cells that carried a scrambled shRNA sequence, the GPR137-shRNA lentiviruses-infected SK-OV-3 cells resulted in the xenografts with the significantly decreased tumor weight and tumor volume in nude mice (Fig. 7A,B,D,H), and the RAB8A-low expressing A2780 cells consistently revealed the decreased ovarian tumor weight in a mouse model of in situ serous ovarian cancer (Fig. 7E,F,I). In addition, both the GPR137-suppression group and the RAB8A-knockdown group exhibited the lowed mice body weight in comparison to the corresponding control (Fig. 7C,G). On the other hand, silencing of RAB8A led to a significant decrease in the proliferation of tumor cells but a significant elevation in the apoptotic OC cell rate, compared to that in the control tumors (Fig. 7J-M). Moreover, the RAB8A-shRNA-expressing tumor group also showed weakened staining of GLI1 and PTCH1 (Fig. 7N) as well as the downregulated protein expression of GLI1 and PTCH1 (Fig. 7O), which was in contrast to the positive effects of RAB8A-ovexpression on xenograft growth and GLI1 expression in HO8910-derived tumors cells (Fig. 4H-K). Taken together, these data demonstrate that GPR137-RAB8A axis through HH plays an important and positive role in OC progression in vivo.

## Discussion

**Fig. 8:**
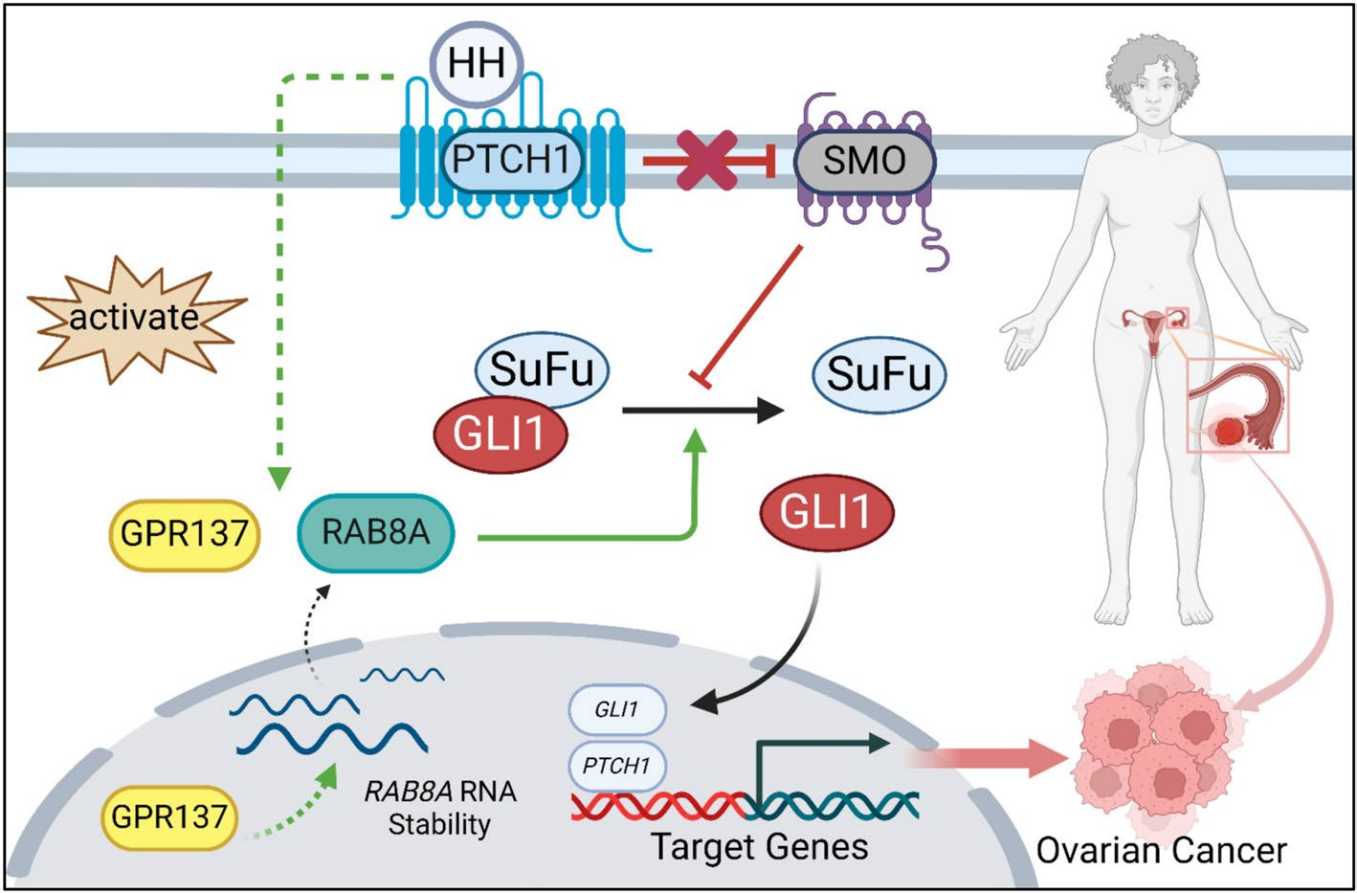
A working model for GPR137/RAB8A/HH/GLI1 signal axis in human OC progression. Created with BioRender.com

In this study, we have confirmed that the orphan receptor, GPR137, acts as a tumor promoter bolstering OC cell propagation through the RAB8A-mediated HH signaling activation. Mechanistically, GPR137 enhances stabilization of *RAB8A* mRNA and the up-regulated RAB8A expression activates HH signaling by destroying the SuFu-GLI1 complex, leading to the release of GLI1 and thereby its translocation into the nucleus to trans-activates cancer-associated target genes (Fig. 8). Therefore, our study depicts the role of the GPR137-RAB8A-HH cascade in the development of OC.

The G protein-coupled receptors (GPRs) family are widely distributed in various tissues and participates in various cell biological processes [37]. As a member of GPR, GPR137 has been shown to contribute to malignant phenotypes of multiple cancers, such as gastric cancer [8], pancreatic cancer [38], bladder cancer [39], and prostate cancer [11]. As previous study showed that, miR-134-3p expression is significantly induced in anisomycin-treated isolated human ovarian cancer tumor stem cells (HuOCSCs), which suppresses the elevated expression of GPR137, one of the genes specifically targeted for regulation by miR-134-3p, and restrains both the in vitro and in vivo activities of HuOCSCs [13], indicating a potential association between the expression levels of GPR137 and the development of OC, and also suggesting that GPR137 would be a promising target for compromise of HuOCSCs activity and a possible prognosis of OC development. In the present study, we have revealed that GPR137 is upregulated in ovarian carcinomas compared to normal non-cancerous ovarian tissues, which is consistent with the GEPIA data and with a previous study [14]. These observations were further attested with different OC cell lines in in vitro experimental assays, showing that ablation of GPR137 impedes, whereas ectopic expression of GPR137 fosters, OC cell growth and biological behaviors including proliferation, migration, invasion and colony formation. Therefore, we have demonstrated that GPR137 could abet OC progression, indicating that suppressing or reversing GPR137 expression would be a potential therapeutic strategy of OC.

The RAB proteins function as scaffolds, connecting signaling pathways and intracellular membrane trafficking processes in different cancers [16]. As a multifunctional GTPase belonging to the Rabs family, RAB8A is involved in the development and metastasis of cancers including female reproductive cancers. Bie et al*.* revealed that the expression of RAB8A was found to be different in endometrial cancer tissues and normal endometrial epithelial tissues, indicating RAB8A might be a new biomarker for endometrial cancer [33], while Liu et al*.* reported that RAB8A promotes the proliferation, migration, and invasion of breast cancer cells through inhibiting Tyrosine Kinase Receptor B (TRKB), a specific receptor for Brain-Derived Neurotrophic Factor (BDNF) [40]. More recently, Ji et al*.* showed that RAB8A was over-expressed in the cervical cancer tissues, and knockdown of RAB8A in cervical cancer HeLa and CaSki cells decreased the cell viability, cloned cells number, and the migrated and invaded cells [32]. On the other hand, as a RAB13 (a member of the Rabs family)-binding protein, the molecules interacting with CasL-like 2 (MICAL-L2) plays an important role in interacting with RAB8A and RAB13 to coordinate the assembly junctions [41]. A previous study reported that, the stronger MICAL-L2 staining was detected in epithelial ovarian cancer (EOC) samples than that in the benign ovarian cysts, which was determined by IHC staining using a tissue microarray that contained 414 EOC tissue samples and 123 benign ovarian cysts [42]. Therefore, it can be supposed that, as the binding partner of MICAL-L2, RAB8A expression is predicted to be higher in EOC than that in benign ovarian cysts and borderline OC samples.

In addition to a previous study showing that, RAB8A in concert with the Ran GTPases regulates nucleo-cytoplasmic shuttling, causing rapid responses to signaling that require changes in ovarian cancer cell growth [43], in this study, we have demonstrated that RAB8A is highly expressed in OC tissues as well as in OC cells, and we have clearly shown that silencing of RAB8A significantly impedes OC cell malignancies both in vitro in different OC cell lines and in vivo in a nude mouse model, enriching the oncogenic function of RAB8A in cancer and also providing with evidence for RAB8A as a promising target for OC intervention and therapy in future. *RAB8A* mRNA expression is found to be regulated by different factors such as transactivation and miRNAs [32, 44], and we herein have uncovered its mRNA stability regulation, making a supplement to the mechanisms on RAB8A expression regulation. While we showed GPR137 modulates *RAB8A* mRNA decay, GPR137 is not directly bound to *RAB8A* mRNA, indicating the involvement of other molecule(s) in this progression regulation, which needs further investigations.

HH signaling play a fundamental role in human development and diseases including cancer [45], such as basal cell carcinomas (BCC) and medulloblastoma [46]. In the absence of HH ligands, GLIs form a protein complex with SuFu, where GLIs (particularly GLI2 and GLI3 proteins) are initially phosphorylated at multiple sites by protein kinase A (PKA) and undergo further phosphorylation by several kinases such as glycogen synthase kinase 3β (GSK-3β) and casein kinase 1 (CK1), leading to their ubiquitination by the β-transducin repeats-containing proteins (β-TRCP) family of E3 ubiquitin ligases and subsequent proteolysis to generate the repressor forms (GLI-R) that “shut-down” HH signaling [47, 48]. In agreement with previous findings, we have shown herein that silencing of RAB8A significantly elevated the GLI3-R levels and accordingly reduced the GLI-Luc activities as well as the expression of HH targets. On the contrary, in response to HH stimulation, GLIs such as GLI1 dissociates from its association with SuFu and subsequently translocates to the nucleus, where GLI1 transactivates target genes such as *MYC*, *Cyclin D1* and *Cyclin D2* as well as *GLI1* and *PTCH1*, which are universally activated in all cell types and function as negative (*PTCH1*) and positive (*GLI1*) feedback mechanisms, respectively [17, 49]. Therefore, the dynamic changes in subcellular localization of GLI1 protein are precisely controlled by the upstream HH signaling, and the upregulation of *GLI1* and *PTCH1* expression thereby serves as a sensitive readout for evaluating mammalian HH pathway activation. Several studies indicate that HH signaling is hyper-activated in OC tissues, and activation of HH signaling promotes OC progression [50–52]. In a study by Liao et al*.*, overexpression of PTCH1 and GLI1 protein in OC is correlated with poor survival of the patients [53]. While GLI1 was mainly distributed in the cytoplasm of ovarian epithelial tumors, a high level of GLI1 expression in invasive cancer samples was associated with scattered nuclear GLI1 immunoreactivity. Moreover, significantly elevated HH activity was observed in OC samples compared to normal ovarian tissues and benign ovarian tumors, and was specific for particular histological types. As shown in a previous study by Chen et al*.*, the protein expression of the key molecules in HH signaling, including Sonic Hedgehog (SHH), Desert Hedgehog (DHH), PTCH1, Smoothened (SMO), and GLI1, was not observed in normal ovarian surface epithelium (OSE), but was increased stepwise in benign, borderline and malignant neoplasms [54], indicating that HH activity is relatively low in benign and borderline OC samples. Consistently, we have proved that HH deactivation significantly decreased OC proliferation, which is in agreement with the GEPIA OC data showing higher expression level of GLI1 is associated with unfavorable prognosis. Similar to the effects of RAB8A-overexpression on the GPR137 silencing-repressed HH activity and OC cell biological behaviors, HH activation effectively reversed the GPR137 knockdown-caused negative effects on OC cell malignancies, reinforcing the notion that GPR137-RAB8A axis mediates ovarian carcinogenesis and progression by controlling HH activity. Intriguingly, the data from human protein ATLAS (https://www.proteinatlas.org/, not shown here) reveal that, *GPR137* is expressed in ovarian stromal cells and endothelial cells but is mainly distributed in oocytes, whereas *RAB8A* expression is largely presented not only in oocytes and mixed immune cells but also in stromal cells, and GLI1 expression is mainly observed in stromal cells. The variable but overlapped expression pattern of these molecules in OC tissues suggest a “relay” role of RAB8A in GPR137 and HH signaling, and also indicate the involvement of other effectors such as immune cells and immune microenvironment in OC progression. Nevertheless, as shown by our RNA-Seq data, the ectopic expression of RAB8A also causes the enrichment of different signalings in addition to the HH, such as the mTOR that commonly functions downstream of the PI3K/AKT signaling axis. Therefore, whether the GPR137-RAB8A axis promotes ovarian carcinogenesis through other signalings is worthy of further explorations in future.

Recent studies have unveiled several effectors of RAB8A and these effectors bind to RBA8A, leading to RAB8A activation and thereby translocation to cell plasma membrane. Sun et al*.* reported that the unconventional motor protein myosin Va (MyoVa) bound to RAB8A and activated RAB8A, demonstrating MyoVa is an effector of Rab8A in muscle [55], whereas Yin et al*.* showed that Berbamine (BBM), a marketing drug for treatment of leukopenia in China, obviously inhibited the activity of RAB8A GTPase in an obesity mouse model [56], indicating the variable activity of RAB8A in different diseases. Owing to the work in the past decades, it has shown that distinct classes of small GTPases are involved in membrane vesicles trafficking, and RAB family proteins are observed to be localized to the surface membrane of different organelle in the cytosolic compartment in certain cells, where they direct a particular membrane trafficking pathway for appropriate protein sorting, targeting, and intercellular communication as well [16, 57]. RAB proteins, including the RAB8A, have been determined to be associated with multiple cancers, and dysregulated interaction between RABs and their effectors could also link to tumor progression and malignancy. The well-known effector proteins, such as guanine nucleotide exchange factors (GEFs), GTPase-activating proteins (GAPs), and guanine dissociation inhibitors (GDIs), control RABs activities, leading to translocation of RABs and further activation/deactivation of the RAB-regulated downstream signalings in certain cancer cells. For example, binding of Formin homology protein interacting protein 2 (FIP2) to RAB11 promotes epithelial-mesenchymal transition (EMT) and metastasis in gastric cancer [58], and this complex also potentiates colorectal cancer cells migration and invasion by inducing MMP-7 expression through activating the PI3K/AKT signaling [59]. In our study, we have identified a potential effector controlling RAB8A activity in ovarian cancer cells. Therefore, further study and develop the small-molecule antagonists targeting RAB8A could hopefully be a promising therapy of ovarian cancer. While we discovered that HH signaling activation triggered the activation of both GPR137 and RAB8A protein, the molecular mechanisms remain yet unknown and further research is thus warranted to elucidate the regulatory mechanisms governing their activities by HH in OC.

In conclusion, our current study has substantiated the critical roles of GPR137-RAB8A axis in HH signaling transduction in OC cells, and the GPR137-RAB8A-HH pathway may provide therapeutic targets for OC.

## Supplementary Information

**Supplementary Material 1: Figure S1**. (A) The protein expression of GPR137 in SK-OV-3 cells transfected with RAB8A shRNA or scrambled shRNA (Con). (B) The protein expression of GPR137 in SK-OV-3 cells transfected with a RAB8A-expressing vector or a control vector (Con). **Figure S2**. (A) A heat-map of normalized expression levels of the altered genes measured by RNA-seq comparing an empty vector-transfected (Control) and a RAB8A-expressing vector-transfected (RAB8A) HO8910 cells. Blue indicates low expression levels and red indicates high expression levels. **Figure S3**. (A) The protein levels of GLI1 and PTCH1 in HO8910 cells transfected with a GPR137-expressing vector (GPR137, +) or a control vector (GPR137, -). **Figure S4**. (A) Matrigel invasion assays of SK-OV-3 (upper) and A2780 (lower) cells transfected with GPR137 shRNA (shGPR137) or scrambled shRNA (Control) in combination with SAG-treatment (SAG, +) or vehicle (SAG, -) for 24 h. Bar, 100 μm. (B) Quantitative analysis of (A, upper: SK-OV-3). (C) Quantitative analysis of (A, lower: A2780). (D) Colony formation assays of SK-OV-3 cells infected with lentiviruses carrying GPR137 shRNA or scrambled shRNA (shRNA-Con) in combination with SAG-treatment (SAG, +) or vehicle (SAG, -). (E) Colony formation assays of A2780 cells infected with lentiviruses carrying GPR137 shRNA or scrambled shRNA (shRNA-Con) in combination with SAG-treatment (SAG, +) or vehicle (SAG, -). (F) Quantitative analysis of relative colony numbers in (D). (G) Quantitative analysis of relative colony numbers in (E). (H) CCK-8 assays of HO8910 cells transfected with a GPR137-expressing vector or an empty vector (Control) in combination with GDC-0449-treatment (GDC) or vehicle and cultured for the indicated time periods. (I) Matrigel invasion assays of HO8910 cells transfected with a GPR137-expressing vector or an empty vector (Control) in combination with GDC-0449-treatment or vehicle for 24 h. Bar, 100 μm. (J) Quantitative analysis of (I). (K) Colony formation assays of HO8910 cells infected with lentiviruses expressing GPR137 or control lentiviruses in combination with GDC-0449-treatment. (L) Quantitative analysis of relative colony numbers in (K). (M) GLI luciferase (GLI-Luc) activities in HO8910 cells transfected with a GPR137-expressing vector or an empty vector (Control) in combination with RAB8A shRNA (shRAB8A, +) or scrambled shRNA (shRAB8A, -). (N) The mRNA levels of *GLI1 *and* PTCH1 *in HO8910 cells transfected with a GPR137-expressing vector or an empty vector (Control) in combination with RAB8A shRNA (shRAB8A, +) or scrambled shRNA (shRAB8A, -). (O) CCK-8 assays of HO8910 cells transfected with a GPR137-expressing vector or an empty vector (Control) in combination with RAB8A shRNA (shRAB8A) or scrambled shRNA and cultured for the indicated time periods. (P) Matrigel invasion assays of HO8910 cells transfected with a GPR137-expressing vector or an empty vector (Control) in combination with RAB8A shRNA (shRAB8A) or scrambled shRNA for 24 h. Bar, 100 μm. (Q) Quantitative analysis of (P). (R) Colony formation assays of HO8910 cells infected with lentiviruses expressing GPR137 or control lentiviruses in combination with lentiviruses expressing carrying RAB8A shRNA (shRAB8A) or scrambled shRNA. (S) Quantitative analysis of relative colony numbers in (R).

## Data Availability

No datasets were generated or analysed during the current study.
