# Supplementary figures and images for: GPR137-RAB8A activation promotes ovarian cancer development via the Hedgehog pathway
Source: J Exp Clin Cancer Res. 2025 Jan 24;44:22. doi: 10.1186/s13046-025-03275-0 (PMC11761205; doi:10.1186/s13046-025-03275-0)

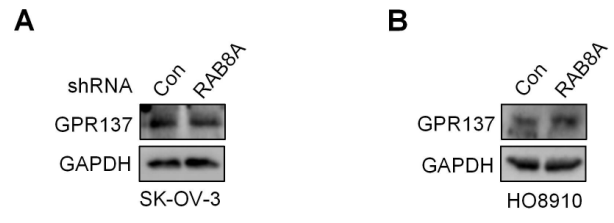

A

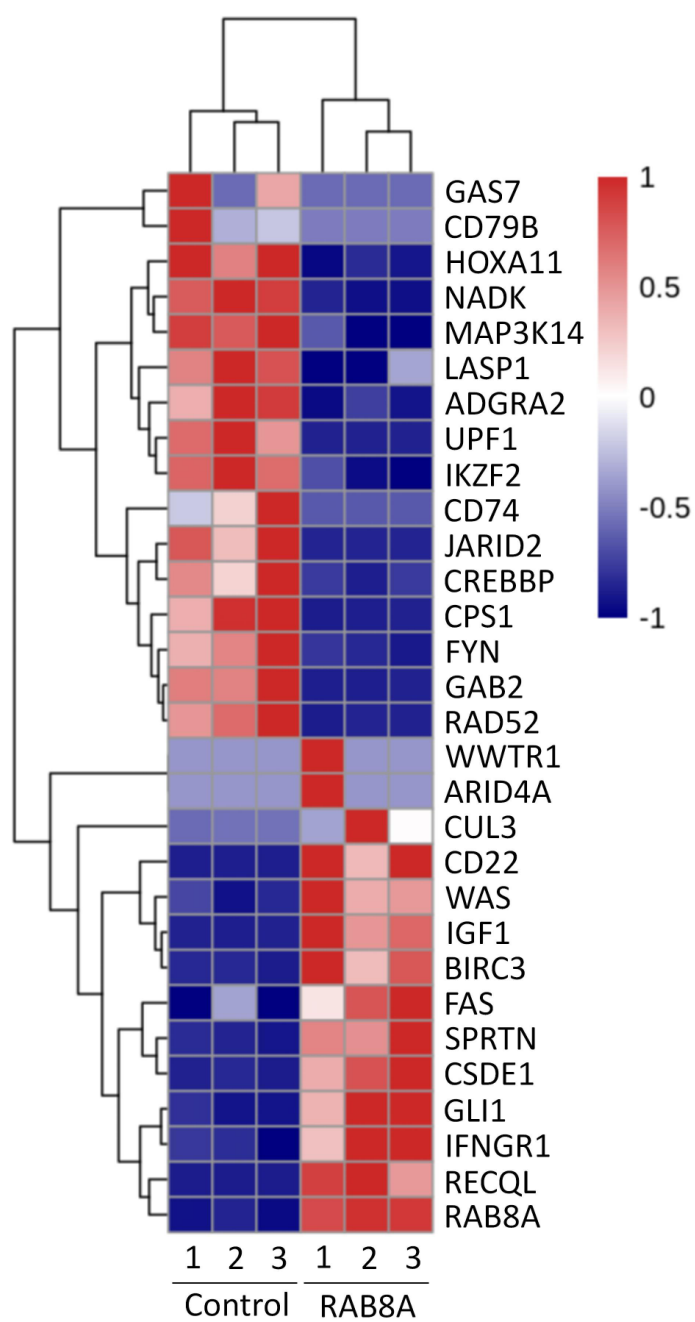

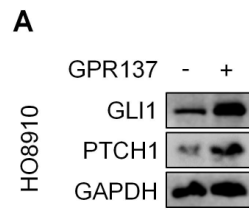

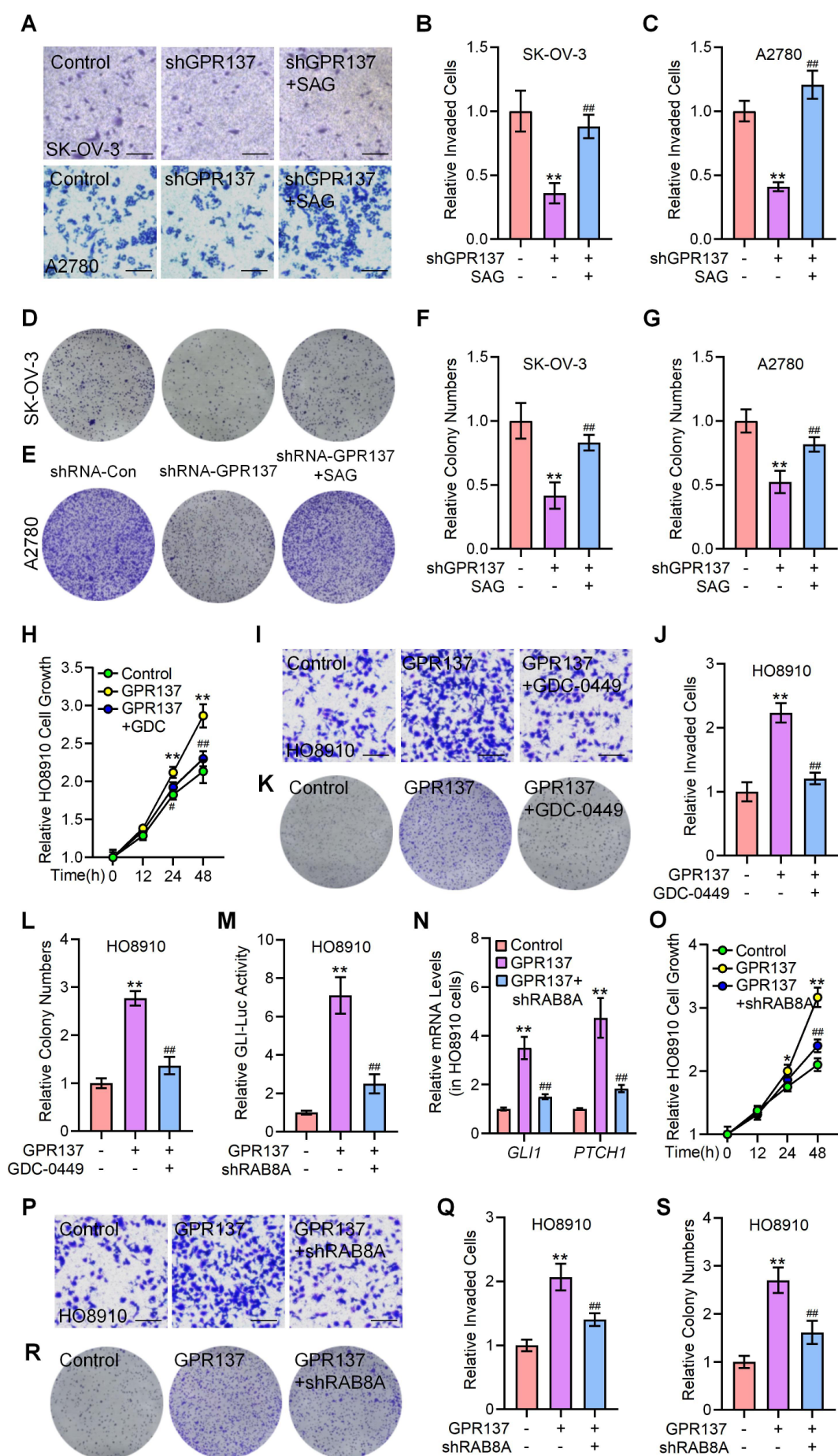

Supplement: Supplementary file 1 — Supplementary Material 1: Figure S1. (A) The protein expression of GPR137 in SK-OV-3 cells transfected with RAB8A shRNA or scrambled shRNA (Con). (B) The protein expression of GPR137 in SK-OV-3 cells transfected with a RAB8A-expressing vector or a control vector (Con). Figure S2. (A) A heat-map of normalized expression levels of the altered genes measured by RNA-seq comparing an empty vector-transfected (Control) and a RAB8A-expressing vector-transfected (RAB8A) HO8910 cells. Blue indicates low expression levels and red indicates high expression levels. Figure S3. (A) The protein levels of GLI1 and PTCH1 in HO8910 cells transfected with a GPR137-expressing vector (GPR137, +) or a control vector (GPR137, -). Figure S4. (A) Matrigel invasion assays of SK-OV-3 (upper) and A2780 (lower) cells transfected with GPR137 shRNA (shGPR137) or scrambled shRNA (Control) in combination with SAG-treatment (SAG, +) or vehicle (SAG, -) for 24 h. Bar, 100 μm. (B) Quantitative analysis of (A, upper: SK-OV-3). (C) Quantitative analysis of (A, lower: A2780). (D) Colony formation assays of SK-OV-3 cells infected with lentiviruses carrying GPR137 shRNA or scrambled shRNA (shRNA-Con) in combination with SAG-treatment (SAG, +) or vehicle (SAG, -). (E) Colony formation assays of A2780 cells infected with lentiviruses carrying GPR137 shRNA or scrambled shRNA (shRNA-Con) in combination with SAG-treatment (SAG, +) or vehicle (SAG, -). (F) Quantitative analysis of relative colony numbers in (D). (G) Quantitative analysis of relative colony numbers in (E). (H) CCK-8 assays of HO8910 cells transfected with a GPR137-expressing vector or an empty vector (Control) in combination with GDC-0449-treatment (GDC) or vehicle and cultured for the indicated time periods. (I) Matrigel invasion assays of HO8910 cells transfected with a GPR137-expressing vector or an empty vector (Control) in combination with GDC-0449-treatment or vehicle for 24 h. Bar, 100 μm. (J) Quantitative analysis of (I). (K) Colony forma [file 13046_2025_3275_MOESM1_ESM.pdf]
